# A comprehensive review of machine learning for heart disease prediction: challenges, trends, ethical considerations, and future directions

**DOI:** 10.3389/frai.2025.1583459

**Published:** 2025-05-13

**Authors:** Raman Kumar, Sarvesh Garg, Rupinder Kaur, M. G. M. Johar, Sehijpal Singh, Soumya V. Menon, Pulkit Kumar, Ali Mohammed Hadi, Shams Abbass Hasson, Jasmina Lozanović

**Affiliations:** ^1^Department of Mechanical and Production Engineering, Guru Nanak Dev Engineering College, Ludhiana, India; ^2^Jadara Research Center, Jadara University, Irbid, Jordan; ^3^Department of Computer Science and Engineering, Guru Nanak Dev Engineering College, Ludhiana, India; ^4^Department of Information Technology, Guru Nanak Dev Engineering College, Ludhiana, India; ^5^Management and Science University, Shah Alam, Malaysia; ^6^Department of Mechanical Engineering, Graphic Era (Deemed to be University), Dehradun, India; ^7^Department of Chemistry and Biochemistry, School of Sciences, JAIN (Deemed to be University), Bangalore, India; ^8^Department of Electrical Engineering, Chandigarh University, Mohali, India; ^9^Chitkara University Institute of Engineering and Technology, Centre for Research Impact & Outcome, Chitkara University, Rajpura, India; ^10^Department of Pharmacy, Mazaya University College, Dhiqar, Iraq; ^11^Laboratories Techniques Department, College of Health and Medical Techniques, Al-Mustaqbal University, Babylon, Iraq; ^12^Department of Engineering, FH Campus Wien - University of Applied Sciences, Vienna, Austria

**Keywords:** heart disease prediction, machine learning (ML), deep learning models, federated learning, explainable artificial intelligence (XAI)

## Abstract

This review provides a thorough and organized overview of machine learning (ML) applications in predicting heart disease, covering technological advancements, challenges, and future prospects. As cardiovascular diseases (CVDs) are the leading cause of global mortality, there is an urgent demand for early and precise diagnostic tools. ML models hold considerable potential by utilizing large-scale healthcare data to enhance predictive diagnostics. To systematically investigate this field, the literature is organized into five thematic categories such as “Heart Disease Detection and Diagnostics,” “Machine Learning Models and Algorithms for Healthcare,” “Feature Engineering and Optimization Techniques,” “Emerging Technologies in Healthcare,” and “Applications of AI Across Diseases and Conditions.” The review incorporates performance benchmarking of various ML models, highlighting that hybrid deep learning (DL) frameworks, e.g., convolutional neural network-long short-term memory (CNN-LSTM) consistently outperform traditional models in terms of sensitivity, specificity, and area under the curve (AUC). Several real-world case studies are presented to demonstrate the successful deployment of ML models in clinical and wearable settings. This review showcases the progression of ML approaches from traditional classifiers to hybrid DL structures and federated learning (FL) frameworks. It also discusses ethical issues, dataset limitations, and model transparency. The conclusions provide important insights for the development of artificial intelligence (AI) powered, clinically applicable heart disease prediction systems.

## 1 Introduction

### 1.1 Background of the study

Cardiovascular diseases (CVDs) cause around 17.9 million deaths each year, accounting for 32% of deaths worldwide. Heart disease continues to be one of the most significant health problems globally and nationally, as it is the leading cause of death around the globe and in the US. In 2021 alone, coronary heart disease was accountable for approximately 9 million deaths. In the US, coronary heart disease caused 1 out of 5 deaths in 2022, affecting all genders and races. The magnitude of this issue is enormous; in the United States alone, heart disease caused approximately two hundred and 52.2 billion dollars in direct and indirect costs from 2019 to 2020 (Khan Minhas et al., 2024). The prevalence of CVDs in the US is anticipated to increase sharply, as 61% of adults are expected to be hypertensive by 2050. The worldwide burden of CVDs is expected to rise by 90% from 2025 to 2050, increasing the number of deaths from 20.5 million in 2025 to 35.6 million by 2050. Therefore, immediate attention needs to be put towards effective heart disease preventive measures, greater detection capabilities, and fairness in healthcare access (Roth et al., 2020; Al-Ajlouni et al., 2024).

**Figure 1:**
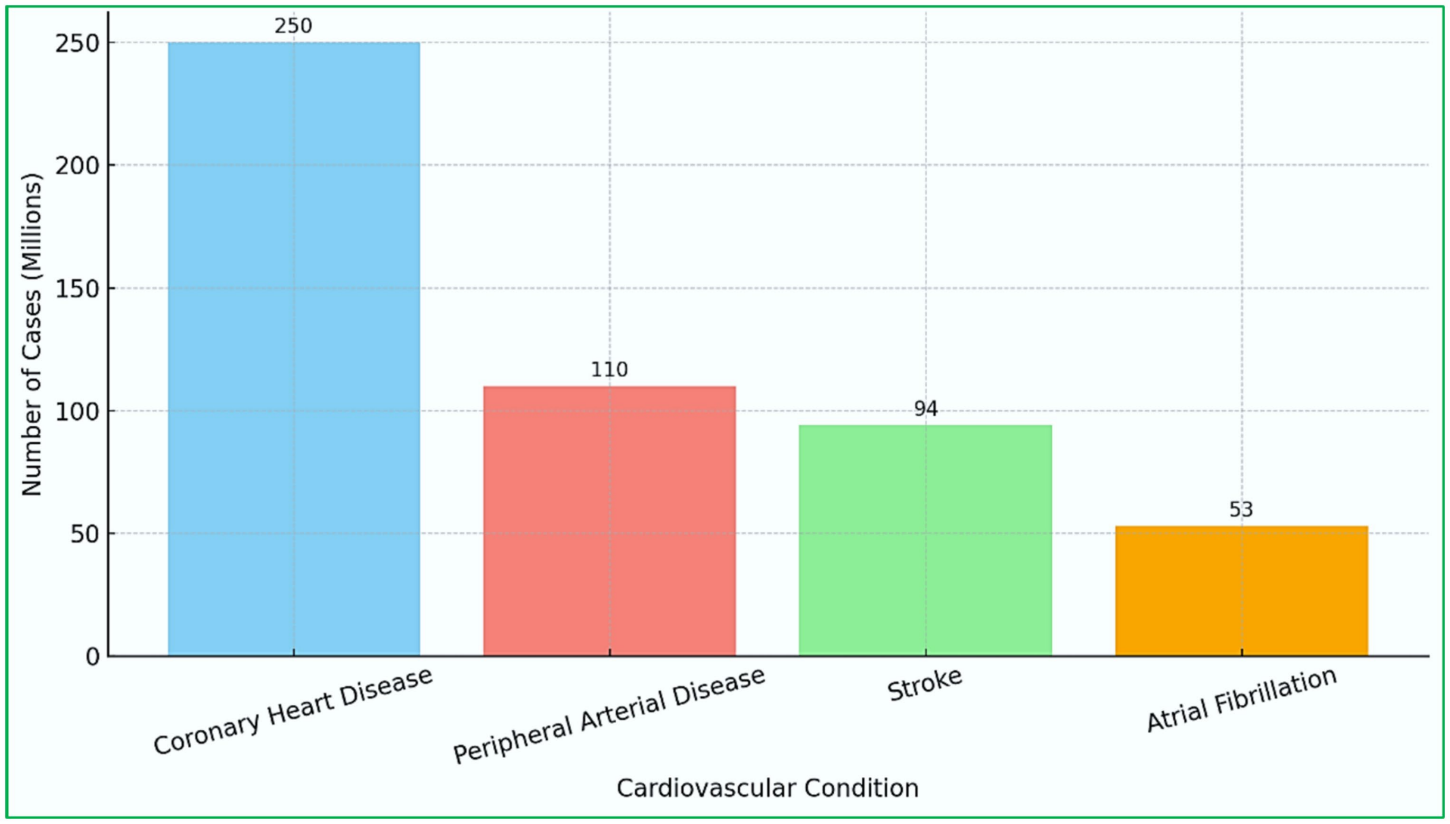
Global prevalence of major cardiovascular diseases.

Many patients can be kept alive through effective healthcare interventions. This, however, requires early detection (Ferdous Azam and Sarwar, 2023). By taking proactive measures, one can help alleviate the bad consequences of the disease, improve the possible prognosis, and save money to be spent on treating the problem. Unfortunately, most diagnostic methods, such as Electrocardiogram (ECG), echocardiograms, and stress testing, need considerable time and skill to administer, and even then, accurate diagnosis may still not be achieved (Dala Ali et al., 2023; Faraji et al., 2023). Such limitations are even more pronounced in underdeveloped areas where such facilities are hard to come by. Machine Learning (ML), a subfield of artificial intelligence (AI), provides solutions to such problems (Pathirana et al., 2018; Pathirana et al., 2019). Complex ML algorithms can recognize intricate structures and correlations existing within a vast data set that are not readily available using traditional techniques. Such an attribute enables chronic diseases of the heart to be diagnosed at intervals much earlier than is possible when patients start showing symptoms. Thus, ML can facilitate the adoption of preventive measures and strides towards a patient-centered approach. Further, ML gives global health a powerful tool for applying affordable and efficient diagnostic technology to populations that need it most (Naruka et al., 2022). Figure 1 depicts the worldwide prevalence (in millions) of major cardiovascular conditions as of 2021. Coronary heart disease remains the most prevalent, impacting roughly 250 million people, followed by peripheral arterial disease (110 million), stroke (94 million), and atrial fibrillation (53 million) (Jagannathan et al., 2019). These figures highlight the significant global challenge posed by CVDs and emphasize the urgent need for effective predictive models driven by ML and AI to facilitate early diagnosis and prompt intervention. Incorporating these technologies into healthcare systems can significantly reduce mortality and enhance patient outcomes.

### 1.2 Role of machine learning in healthcare

The adoption of electronic health records (EHRs) and wearable devices, and sophisticated imaging technologies is aiding the healthcare industry in data management (Bai and Mardini, 2024). The ability of ML to use such data to improve clinical processes and patient interaction is astounding. In predicting heart disease, diverse data sources are harnessed by ML models such as (Pathirana et al., 2019; Dissanayake and Johar, 2023):

- Clinical Data: Data about the patient that includes demographics, medical history, lab results, and medications.
- Imaging Data: Echocardiograms, angiograms, and computed tomography (CT) scans.
- Biometric Signals include ECG, heart rate variability, and blood pressure.
- Data from wearable devices includes daily physical activities, sleep patterns, and vital signs.

**Figure 2:**
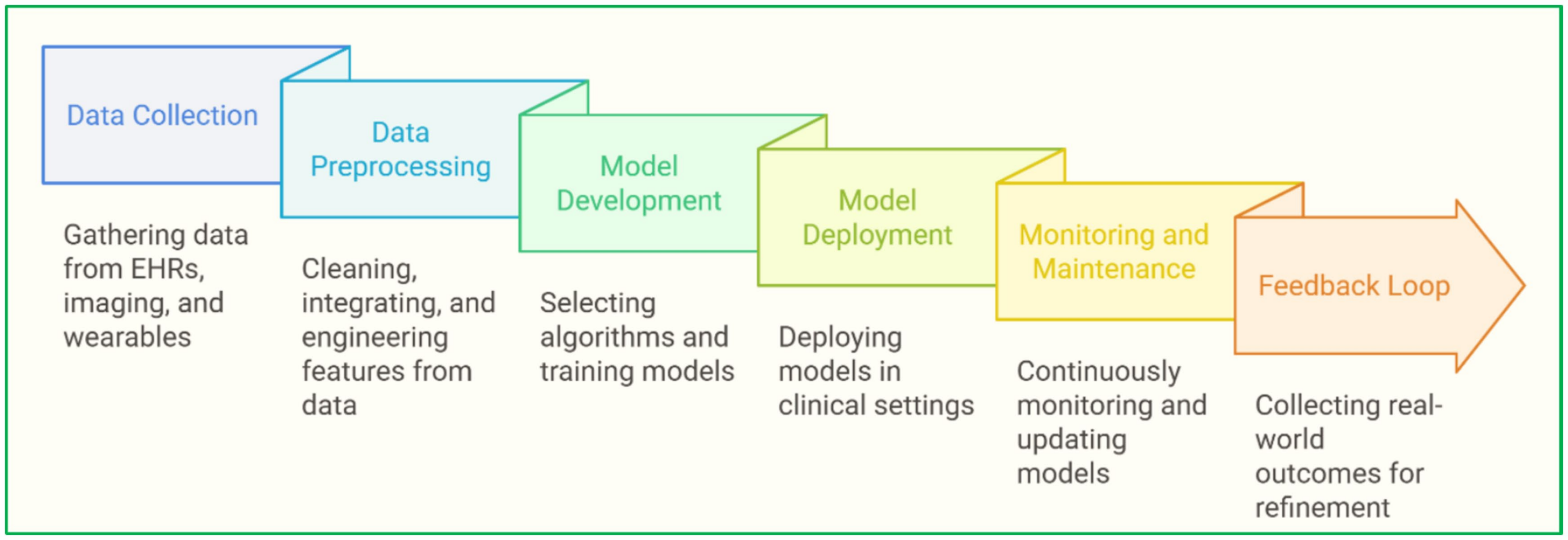
ML in heart disease prediction, from data collection to model deployment.

**Figure 3:**
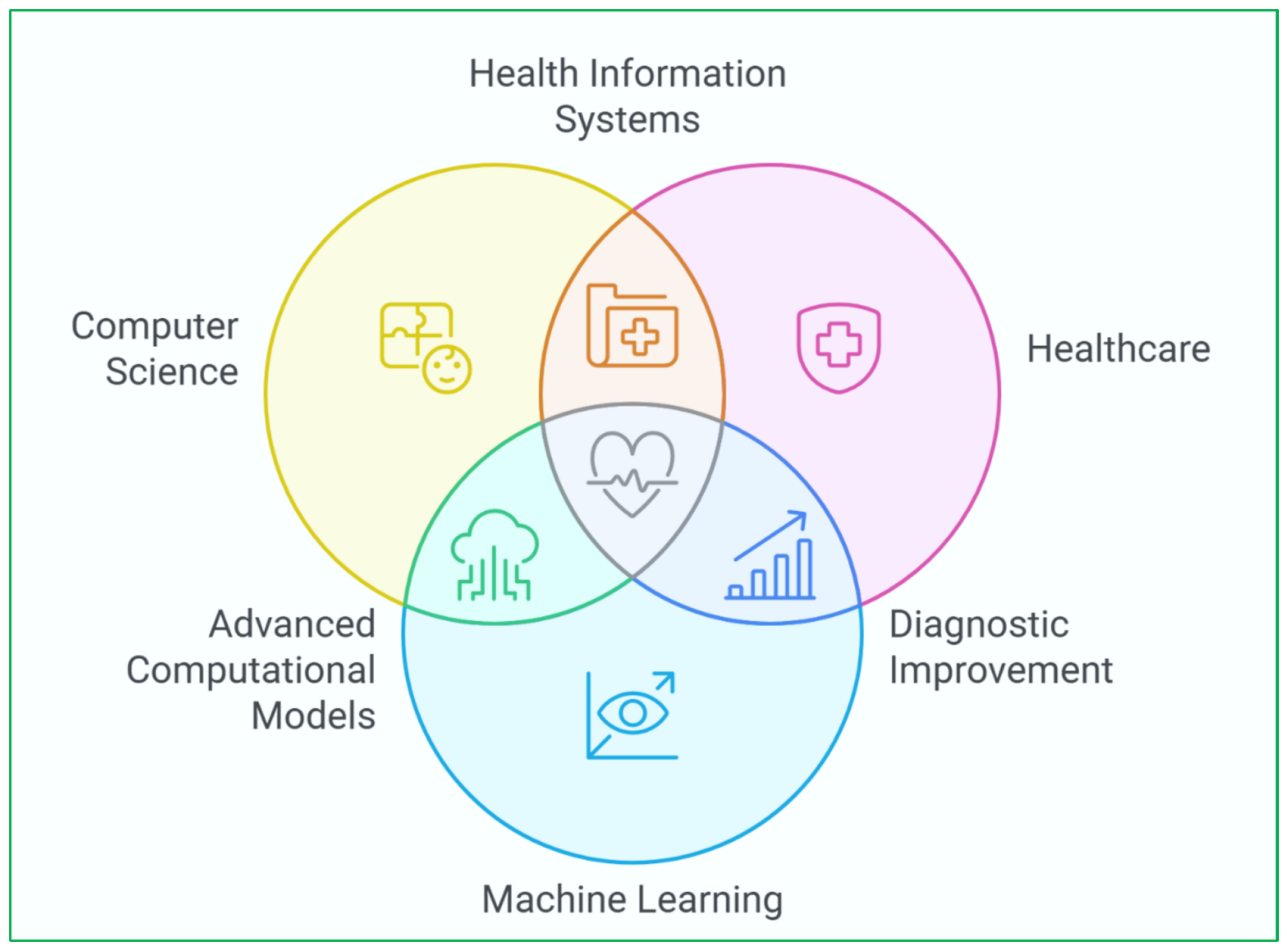
Integrated heart disease prediction.

Devices that classify people as high risk and low risk of heart disease are based on supervised learning techniques like random forest (RF), support vector machine (SVM), and neural network (NN). Slicers of the ML set are DL, which enhances a machine’s learning capabilities. For instance, convolutional neural network (CNN) has been used to identify arrhythmias from ECG signals with great precision. Furthermore, clustering techniques consider the unsupervised learning approach in which algorithms detect groups within a patient population that can be linked to specific risk levels or responses to treatment, thus paving the way for targeted medicine (Dissanayake and Johar, 2021; Dissanayake et al., 2023). Another innovative area is the application of reinforcement learning (RL) to improve treatment plans and the allocation of resources in healthcare settings. Figure 2 depicts ML in heart disease prediction, from data collection to model deployment (Nadeem et al., 2021; Kwon and Dong, 2022). Figure 3 presents an integrated heart disease prediction (Siramshetty et al., 2018; Benhar et al., 2020).

### 1.3 Objectives and scope of the review

The review aims to incorporate findings from previous research studies on heart diseases while creating, developing, and applying ML technologies that predict heart diseases. Grouping these studies into thematic clusters may help understand the advancements in the field, highlight strengths and challenges, and lay out the following objectives.

- *Categorization of Research:* Formulating a primary information scheme by grouping the studies into five main clusters.
- *Analysis of ML Models:* Performed in-depth analysis of the models in terms of algorithms, techniques, components of the systems, and their merits and demerits alongside real-world applications.
- *Feature Engineering and Optimization:* Striving to improve model performance through feature selection, dimensionality reduction, and hyperparameter tuning.
- *Emerging Technologies:* Exploiting the effects of innovations such as FL, quantum computing (QC), and Internet of Things (IoT) devices on the diagnostics of heart diseases.
- *Other Applications of AI:* AI’s involvement in treating diseases like cancer, diabetes, and other neurological disorders should be emphasized to better understand how heart disease prediction can be approached.

This review proposes to find patterns, gaps, and trends by incorporating these clusters in literature patterns to provide better insights for evolving studies and their implementations.

### 1.4 Comparison with existing literature and novel contributions

There is a substantial body of literature on ML applications in the healthcare sector, with review articles examining ML usage in healthcare diagnoses, predictions, and treatments. The literature is extensive. While many studies have been conducted, a gap remains in the focus on heart disease prediction through ML systems. We directly compare what this review achieves to existing works to address this gap.

#### 1.4.1 Comparative analysis of related reviews

To illustrate how this review differentiates from prior works, Table 1 compares key literature, focusing on scope, methodology, datasets, and technological advancements.

**Table 1:** Comparison of existing literature on ML for heart disease prediction.

References	Scope	Methodology	Datasets used	Technological focus	Challenges discussed	Novel contributions
Hajiarbabi (2024)	Overview of ML for CVDs	A systematic review of ML models	UCI Heart Disease, Framingham, PhysioNet	DL and supervised learning	Model performance and dataset limitations	Lacks discussion on ethical challenges and explainability
Ahsan and Siddique (2022)	ML applications in healthcare	Meta-analysis of diagnostic accuracy	Multiple EHR datasets	Traditional ML SVM, RF, K-nearest neighbor (KNN)	Data imbalance and interpretability	It does not explore FL or QC
Jafari et al. (2023)	DL models for ECG-based heart disease detection	Experimental comparison	Physikalisch-Technische Bundesanstalt Extended ECG Dataset (PTB-XL), MIT-BIH, ECG datasets	CNN, LSTM, Transfer Learning	Bias, data augmentation	Lacks coverage on FL and real-world integration
Present review	Comprehensive review of ML for heart disease prediction	Thematic classification with clustering	EHR, ECG, Cleveland, Framingham, Emerging IoT-based datasets	Supervised and unsupervised learning, DL, FL, QC	Ethical concerns, dataset quality, integration into clinical practice	Integrates AI trends (IoT, FL, QC) and highlights regulatory, transparency, and privacy challenges in ML adoption

#### 1.4.2 Novel contributions of this review

Though other reviews provide essential insights into ML-based heart disease prediction, they tend to suffer from some drawbacks. Unlike other works, our work is unique in the following aspects.

##### 1.4.2.1 Newer dataset analysis

○ This review covers newer datasets such as IoT-based healthcare data, wearable sensor datasets, and FL-enabled datasets, unlike other reviews focusing on classical datasets like UCI heart disease data and Framingham data.
○ Covers real regulatory challenges such as imbalance and noise.

##### 1.4.2.2 Emerging technology integration

This review deeply analyzes new emerging AI tools like:

○ FL—privacy-preserving ML.
○ QC—high-speed disease modeling.
○ Explainable AI (XAI)—model understanding and trust.
○ Other reviews hardly address the confluence of AI with regulatory frameworks like General Data Protection Regulation (GDPR), Food and Drug Administration (FDA), and Health Insurance Portability and Accountability Act (HIPAA).

##### 1.4.2.3 Ethical and regulatory considerations

○ Covers biases and patient privacy ML in healthcare ethics issues and explainability.
○ Provides an in-depth discussion of the legal aspects of AI implementation in healthcare.

##### 1.4.2.4 Structured thematic classification

○ Unlike other systematic reviews, this piece of work classifies and indexes research into 5 different thematic clusters.
○ Methods and Algorithms of Heart Disease Detection and Diagnostics.
○ ML Models and Algorithms.
○ Emerging Feature Molding and Engineering.
○ Advanced Emerging Technology.
○ Multi-Disease AI Technology Applications.

##### 1.4.2.5 Bridging the gap between research and clinical application

○ Prior works focus primarily on ML model accuracy, while this review focuses on actual clinical application.
○ Explains how different hospitals, medical practitioners, and policymakers can use ML-based systems for diagnosis in real-life settings.

#### 1.4.3 Heatmap provides feature correlations

**Figure 4:**
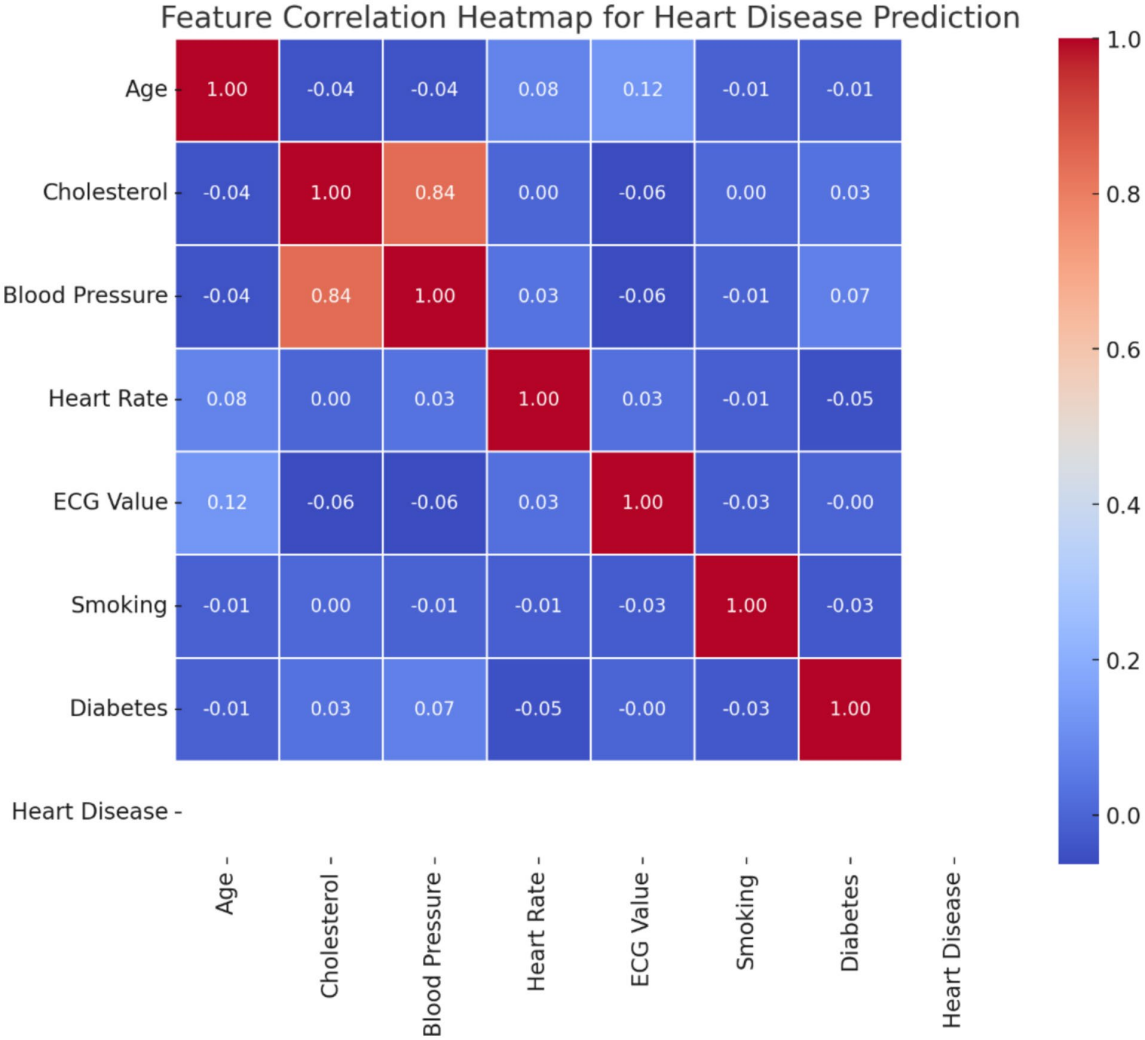
Feature correlation heatmap for heart disease prediction.

A heatmap is used in a dataset with heart disease features to represent the relationships of different attributes. Redundancy may exist for the features with high correlations, such as cholesterol and blood pressure, but weakly correlated features suggest that they would be independent in their ability to predict. Figure 4 presents a feature correlation heatmap that depicts the relationships between essential clinical variables like cholesterol, blood pressure, and age, which are vital for enhancing feature selection in heart disease prediction models.

- Proposition: ‘Blood Pressure and Cholesterol,’ a high correlation suggests potential multicollinearity and multivariate relationships.
- Counter Proposition: ‘Age and Resting ECG,’ the low correlation suggests a weak direct association.
- Supporting Proposition: ‘Smoking and Heart disease’ have a moderate correlation, which supports known medical findings.

## 2 Methodology

### 2.1 Literature search and inclusion criteria

The review methodology entailed a comprehensive literature search. The search involved a comprehensive query in Scopus using the search string:

((TITLE-ABS-KEY (‘heart disease prediction’) AND (‘ML’) OR (‘machine learning’)) AND (LIMIT-TO (DOCTYPE, “ar”)) AND (LIMIT-TO (LANGUAGE, “English”))).

It filtered relevant articles on heart disease prediction using ML, limited to English-language articles and journal articles. The curated results form the foundation for the insights and analyses presented in this review. Studies were excluded if they lacked adequate methodological detail, were not peer-reviewed, or were focused on unrelated topics. Quantitative and qualitative insights were extracted to identify trends, assess proposed methods’ effectiveness, and uncover existing research gaps.

### 2.2 Classification of clusters

**Figure 5:**
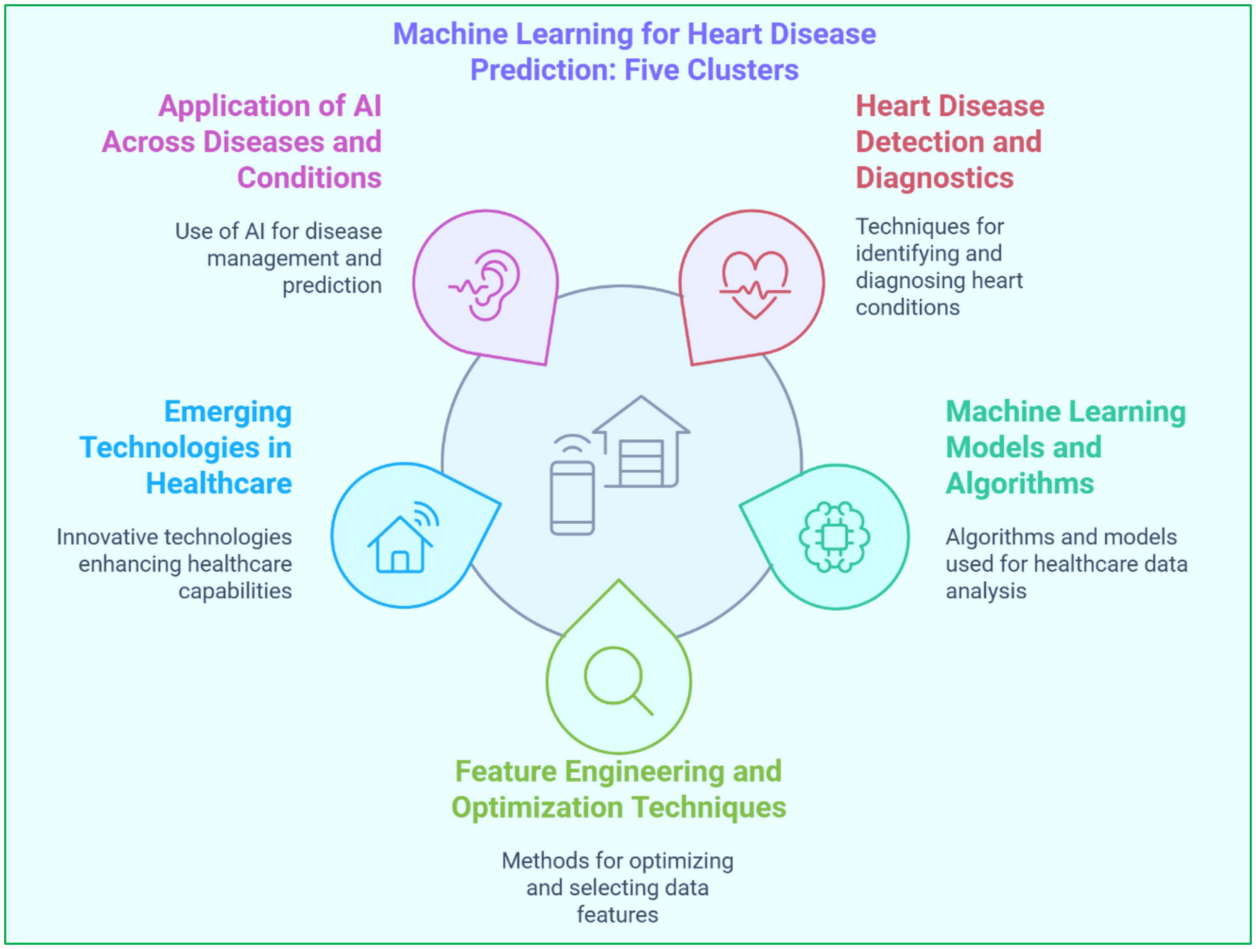
Machine learning for heart disease prediction: five clusters.

The identified studies have been classified into five major clusters based on their primary focus using keywords. Table 2 depicts keywords considered and cluster names based on keywords and the occurrence of keywords. The first cluster, ‘*Heart Disease Detection and Diagnostics*’, includes advanced research on diagnostics and predictions concerning heart-related conditions. The second cluster, ‘*Machine Learning Models and Algorithms for Healthcare*,’ studies the different types of ML model frameworks, including supervised and DL models. The third cluster, ‘*Feature Engineering and Optimization Techniques*,’ discusses papers on feature selection, dimensionality reduction, and other optimization methods in model performance improvement. The fourth cluster, ‘*Emerging Technologies in Healthcare*’, reviews articles on modern innovations like QC, FL, and IoT in heart disease prediction and other applications. The last or fifth cluster, ‘*Applications of AI Across Diseases and Conditions*,’ gives more context by including AI’s applications in managing and predicting other diseases such as cancer, diabetes, and neurological disorders. This categorization is shown in Figure 5, facilitating a more profound literature review and allowing a better understanding of each cluster’s contributions and limitations.

**Table 2:** Clusters of ML for heart disease prediction.

Cluster number	Cluster name	Keywords	Occurrence
1	Heart Disease Detection and Diagnostics	Heart disease classification, heart disease detection, Heart failure, Arrhythmia, Valvular heart disease, Electrocardiogram (ECG), PCG signal analysis, Cleveland and Framingham Datasets, Acute Myocardial Infarction, Cardiac disease	12
2	Machine Learning Models and Algorithms for Healthcare	Machine learning, Artificial intelligence, Deep learning, Logistic Regression, Decision tree, Support vector machine (SVM), Random Forest classifier, Gradient descent, Sparse autoencoder, Attention mechanisms, Transformer-based models, Recurrent neural network (RNN)	13
3	Feature Engineering and Optimization Techniques	Feature extraction, Feature selection, Principal component analysis (PCA), SHAP, Genetic Algorithm (GA), Grey wolf algorithm, Particle Swarm Optimization (PSO), Sand Cat Swarm Optimization, Coati optimization algorithm, Kepler optimization, Canonical Correlation, Lasso regression	12
4	Emerging Technologies in Healthcare	Healthcare 4.0, Quantum computing, Federated learning, 5G, Wearable devices, Internet of Things (IoT), Cloud platform, Automated Sequential Cryptography, Cloud security, Decryption, Firefly Algorithm, Modified Blowfish	10
5	Applications of AI Across Diseases and Conditions	Diabetes, Diabetic retinopathy, Breast cancer, Parkinson’s disease, Stroke prediction, cardiovascular diseases (CVD), Disease Prediction, Early Diagnosis, Scalability in machine learning, Multimodal feature fusion, Cross-modal transfer learning	11

## 3 Cluster wise insights: literature review

### 3.1 Cluster 1: heart disease detection and diagnostics

**Figure 6:**
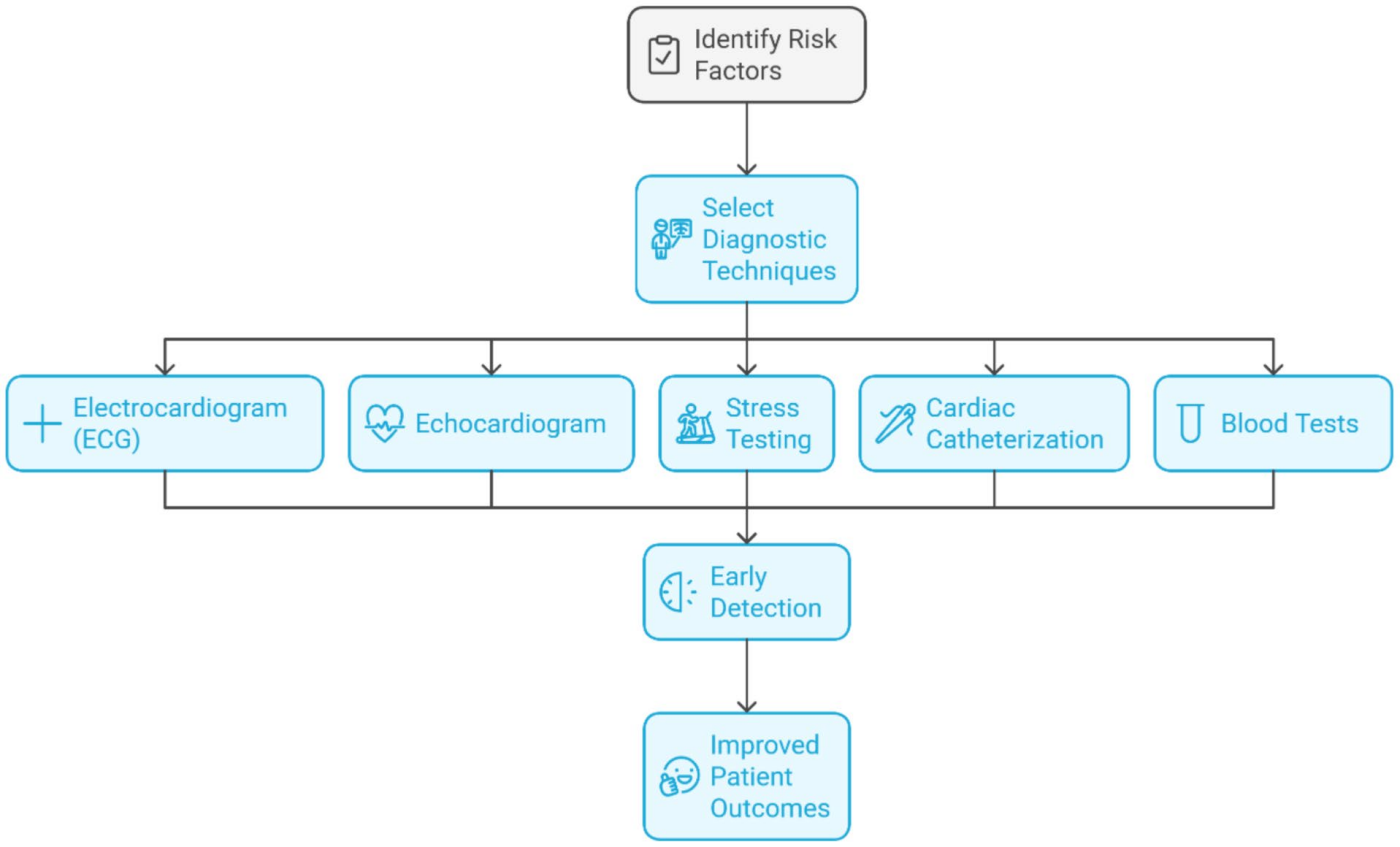
Diagnostic techniques and multidimensional approach to heart disease management.

Technological advancements and methodologies provide innovative approaches and tools for the early detection and prediction of cardiovascular ailments. This group focuses on new imaging and ML model development, which improves diagnostic precision and appropriate timing of interventions to improve the prognosis for patients. Heart disease displays itself as more than just coronary artery disease (CAD); it includes arrhythmias and even heart failure. Therefore, it is essential to stress the multi-dimensional approach to conditions related to heart diseases. As outlined in Figure 6, critical steps are monitoring ECG for electrical activity, echocardiograms for the anatomical view, stress tests for function, cardiac catheterization for the coronary details, and blood tests for the troponin marker, along with the cholesterol marker, which are the most important for attention.

Table 3 shows key insights and performance metrics in heart disease detection and diagnostics. The classification of heart disease proved accurate with DL algorithms and Sand Cat Swarm Optimization (SCSO) for feature selection. Important features were identified using patient pathology data, and models, including CNN, PCA, Restricted Boltzmann Machine (RBM), and deep convolutional generative adversarial networks (DCGAN), analyzed intricate correlations that improved the accuracy of predictions. The method enhanced the reliability of heart disease prognosis through metrics such as accuracy and F1-score (Baviskar et al., 2023). The detection of CVD was improved using both RF and eXtreme Gradient Boosting (XGB) on ECG datasets, particularly Physionet 2016, PASCAL, and MIT-BIH. Pre-processing feature extraction with empirical wavelet transform (EWT), discrete wavelet transform (DWT), and SHapley Additive exPlanations (SHAP) will improve a prediction model’s accuracy, and a significant peak can reach up to 98.25 AUC for XGB-based proposed models (Majhi and Kashyap, 2024). A novel Wolf-based Generative Adversarial System (WbGAS), was developed to classify heart diseases, using ECG data to identify normal sinus rhythm, arrhythmia, and congestive heart failure (Goud et al., 2024). A hyperparameter-tuned CNN-based Inception Network model was created to diagnose heart disorders with heart sound data from standard repositories. The model achieves 99.65% accuracy, 98.8% sensitivity, and 98.2% specificity, surpassing the other classifiers (Roy et al., 2024). An electronic stethoscope has been developed, integrated with Raspberry Pi 4B and a CNN-based EfficientNet-B3 model for diagnosing valvular heart diseases. The system reported an accuracy of 99.35%, sensitivity of 98.84%, and specificity of 98.23%, with real-time Phonocardiogram (PCG) signal analysis and cloud-based data storage (Roy et al., 2023). Fatima and Siddiqi (2024) evaluated ML techniques for predicting Myocardial Infarction (MI) and analyzing risk factors using a dataset of 350 individuals, including MI and non-MI patients of both genders.

**Table 3:** Key insights and performance metrics: cluster 1 ‘heart disease detection and diagnostics’.

Research focus	Key methods/models	Dataset(s) used	Performance metrics	Key insights	Ref.
Heart Disease Detection with SCSO	DL + SCSO, CNN, PCA, RBM, DCGAN	Patient Pathology Data	High Accuracy, Improved F1-score	Enhanced feature selection and reliable prognosis	Baviskar et al. (2023)
ECG Analysis with RF and XGB	RF, XGB, EWT, DWT, SHAP	Physionet 2016, PASCAL, MIT-BIH	Up to 98.25 AUC	Effective feature extraction and prediction	Majhi and Kashyap (2024)
WbGAS for Heart Disease Classification	Wolf-based Generative Adversarial System (WbGAS), Wolf Fitness Function	ECG Data	High Specificity, Precision, Recall, and Accuracy	Outperforms traditional ML methods	Goud et al. (2024)
Hyperparameter-tuned CNN Inception Network	CNN-based Inception Network	Heart Sound Data	99.65% Accuracy, 98.8% Sensitivity, 98.2% Specificity	Efficient handling of high-dimensional data	Roy et al. (2024)
Myocardial Infarction Prediction	Ensemble Classifiers	Dataset of 350 Individuals (MI & Non-MI)	Improved Gender-Specific Precision, High Accuracy	Optimized early MI detection	Fatima and Siddiqi (2024)
Multimodal Data Analysis with CNNs	CNN, Python	BMI, ECG, PTB	98% Accuracy	Reliable prediction from multimodal data	Maran (2023)
Autoencoder + DenseNet on UCI Cleveland Dataset	Autoencoder, DenseNet	UCI Cleveland	99.67% Mean Accuracy, 99.99% Test Accuracy	Outstanding performance requires more	Saleh Alghamdi et al. (2024)
CNN-Bi-LSTM with Attention Mechanisms	CNN-Bi-LSTM, Newton–Raphson Optimizer	Cleveland, Framingham	95.3% Accuracy (Cleveland), 98.1% Accuracy (Framingham)	Optimized cardiac disease prediction	Kayalvizhi et al. (2024)

Maran (2023) developed advanced CNN-based models to analyze multimodal data, including BMI, ECG, and PTB, achieving 98% accuracy in predicting heart diseases. The models were trained on 80% of the data, validated on 20%. Saleh Alghamdi et al. (2024) proposed a method by integrating autoencoder and DenseNet architectures to predict heart disease based on the UCI Cleveland dataset, obtaining a mean accuracy of 99.67% and test accuracy of 99.99%.

### 3.2 Cluster 2: machine learning models and algorithms for healthcare

Table 4 shows Advances in ML Models and Algorithms for Healthcare. A three-stage wireless body area network (WBAN)-based heart disease prediction model was developed. The three stages include data aggregation, channel selection, and prediction. Channel selection was optimized using the Tunicate Swarm-Sail Fish Optimization algorithm, and statistical features were extracted from the data using a weighted entropy-based method. An enhanced RNN tuned with Tunicate Swarm-Sail Fish Optimization achieved high prediction accuracy (Muthu Ganesh and Nithiyanantham, 2022). This research developed ML models to predict cancer, diabetes, diabetic retinopathy, and heart-related outcomes using EHRs. SVMs achieved 97.08 and 79.75% accuracy for cancer and Pima diabetes datasets, respectively, while Decision Tree (DT) reached 86.42% for heart-related predictions. The study showed that ML could enhance disease prediction and patient outcomes (Sheik Abdullah et al., 2024).

**Table 4:** Advances in ML models and algorithms for healthcare.

Research focus	Key methods	Dataset(s) used	Performance metrics	Key insights	Ref.
WBAN-based Heart Disease Prediction	Tunicate Swarm-Sail Fish Optimization, RNN, Weighted Entropy-based Features	WBAN Data	High Prediction Accuracy	Optimized channel selection improves prediction performance	Muthu Ganesh and Nithiyanantham (2022)
ML for Cancer, Diabetes, and Heart Disease Prediction	SVM, DT	Cancer, Pima Diabetes, and Heart Disease Datasets	97.08% (Cancer), 79.75% (Diabetes), 86.42% (heart disease)	ML enhances disease prediction and patient outcomes	Sheik Abdullah et al. (2024)
Real-Time Data Prediction Using ML	DT, NB, RF, KNN, NN	Custom Dataset (300 Instances, 14 Attributes)	Performance Varies Across Classifiers	Useful in predicting heart disease using real-time attributes	Jayakiran et al. (2019)
Chronic Heart Disease Prediction with RFE	RFE, KNN, DT	Cleveland Hungarian CHD Dataset	89.91% Accuracy	RFE improves early CHD prediction	Napa et al. (2024)
ANN and LR for Disease Prediction	ANN, LR, Scaling Methods	Heart Disease Dataset	86.13% (Without Scaling), 98.81% (With Scaling)	Scaling methods enhance ANN performance	Seeli and Thanammal (2024)
IoT and ML for Heart Disease Prediction	IoT Sensors, Hybrid Feature Extraction, RNN, SVM, NB, RF	Sensor Data (BP, Oxygen, EEG)	Higher Accuracy with RNN	Reliable for heart disease detection and classification	Patil et al. (2023)

The real-time data will be utilized to predict heart disease with the support of the ML algorithm, as well as attributes like BP, sugar, and heartbeat. The attributes are applied in the dataset on 300 instances with 14. It is used to train and test an R model. Accuracy Evaluation: Accuracy is measured as the basis of several classifiers (Jayakiran et al., 2019). Napa et al. (2024) evaluated recursive feature elimination (RFE) for classifying chronic heart disease based on the Cleveland Hungarian CHD dataset. Different methods of supervised learning have been tested. The KNN model and the DT attained 89.91%. Seeli and Thanammal (2024) explored ML algorithms’ performance, specifically artificial neural network (ANN) and logistic regression (LR) for disease prediction. Without scaling, ANN obtained 86.13% accuracy in the heart disease prediction, and when ensemble normalization and standardization were applied, the improvement in ANN accuracy was 98.81%. Patil et al. (2023) presented a prediction model for heart disease incorporating IoT and ML techniques using data from several sensors: BP monitors, blood oxygen sensors, and EEGs. Hybrid feature extraction methods were combined with ML algorithms, where RNN yields higher accuracy than the traditional approach using methods like SVM, Naive Bayes (NB), and RF.

### 3.3 Cluster 3: feature engineering and optimization techniques

Table 5 shows Feature Engineering and Optimization Techniques in Heart Disease Prediction. The study proposed a method that combines PCA and feature selection to reduce the dimensionality of data and improve the prediction of coronary heart disease. The model using classifiers such as PCA, RF, DT, and AdaBoost achieved 96% accuracy and outperformed traditional precision, recall, and AUC models. The approach effectively enhanced CHD prediction and patient outcomes (Cheekati et al., 2024). Gupta et al. (2022) proposed a computational intelligence system, C-CADZ, for diagnosing CAD using the Z-Alizadeh Sani CAD dataset. The applied feature extraction, Synthetic Minority Over-sampling Technique (SMOTE) for dealing with class imbalance, and ML classifiers such as RF and Extra Trees resulted in an accuracy of 97.37%. C-CADZ outperformed prior methods by 5.17% and exhibited robust performance, thus making it applicable for heart disease predictions.

**Table 5:** Cluster 3 feature engineering and optimization techniques in heart disease prediction.

Research focus	Key methods/models	Dataset(s) used	Performance metrics	Key insights	Ref.
PCA and Feature Selection for CHD Prediction	PCA, RF, DT, AdaBoost	Coronary Heart Disease Data	96% Accuracy	PCA and feature selection improved precision, recall, and AUC for CHD	Cheekati et al. (2024)
C-CADZ System for CAD Diagnosis	Feature Extraction, SMOTE, RF, Extra Trees	Z-Alizadeh Sani CAD Dataset	97.37% Accuracy	Outperformed prior methods by 5.17%, robust performance for heart disease prediction	Gupta et al. (2022)
Diabetes Detection Using Optimized Classifiers	SVM, KNN, RF, PSO Algorithm for Optimization	Indian Pima Diabetes Dataset	94.27% Detection Rate	Outperformed single classifiers for diabetes prediction	Shimpi et al. (2024)
SMOTE-based Hybrid DL Network for CVD Prediction	SMOTE, Adaptive Coati Optimization, Kepler-Optimized Deep Stacked Recurrent Network	Heat-failure-clinical-records Dataset	95.52% Accuracy	SMOTE-HDL network outperformed existing classifiers	Barfungpa et al. (2024)
DL for Cardiac Disorder Detection	ST-CNN-GAP-5, SHAP Analysis	PTB-XL ECG, Arrhythmia Dataset	93.41% AUC, 95.8% Accuracy, 99.46% AUC	Interpretability with SHAP and better performance than existing models	Anand et al. (2022)
Hybrid CCRF Model for Heart Disease Prediction	Canonical Correlation Analysis, RF, Polynomial Features	–	99.45% Accuracy, Improved Sensitivity, Specificity, Precision, and F1 Score	Maximized feature correlations for heart disease prediction	Vetrithangam et al. (2024)
ALAN Method for Heart Disease Prediction	ANOVA, Lasso Regression, ET-ABDF Model	–	88.0% Accuracy, 89.81% Precision, 96.21% AUC	Superior performance compared to other algorithms	Mandula and Vijaya Kumar (2024)
SCSO and DL for Heart Disease Classification	SCSO, CNN, PCA, GANs	Patient Pathology Data	High Accuracy, Precision, Recall, F1-Score	Enhanced prognosis and reliability in heart disease prediction	Lenin and Venkatasalam (2024)

Shimpi et al. (2024) proposed a model optimized for diabetes detection via SVM, KNN, and RF classifiers with decision-level fusion. The classifiers were optimized utilizing a PSO algorithm, considering clinical data such as age, BMI, blood pressure, and glucose. The model performed at a diabetes detection rate of 94.27%. It outperformed single classifiers and previous methods on the Indian Pima diabetes dataset. Barfungpa et al. (2024) anticipated a new SMOTE-based hybrid DL network for predicting patient survival in CVD. The SMOTE-HDL network, tested on the Heat-failure-clinical-records dataset, obtained a predictive accuracy of 95.52% that outperformed any existing classifiers. Anand et al. (2022) utilized deep NNs on the PTB-XL ECG dataset for cardiac disorder detection, presenting the ST-CNN-GAP-5 model that resulted in an AUC of 93.41%. The model was tested on an arrhythmia dataset and yielded 95.8% accuracy with an AUC of 99.46%, better than other existing approaches. The SHAP analysis showed that the model is interpretable and reveals critical ECG wave changes that can help make diagnoses in resource-constrained environments. Vetrithangam et al. (2024) proposed the Hybrid CCRF model of heart disease prediction, which applied Canonical Correlation Analysis and RF together. The model represented the non-linear relationships and maximized the feature correlations because it generated polynomial features and synthesized canonical variables. Mandula and Vijaya Kumar (2024) proposed the ALAN method, which combines ANOVA and Lasso regression to identify the most essential features for heart disease prediction. The Extra Trees Adaptive Boosted Decision Forest model reached 88.0% accuracy, 89.81% precision, and 96.21% AUC, which is superior to other algorithms. Lenin and Venkatasalam (2024) utilized DL and SCSO for accurate heart disease classification. SCSO selected key features from patient pathology data, enabling CNNs combined with advanced models like PCA and GANs to predict disease severity.

### 3.4 Cluster 4: emerging technologies in healthcare

Table 6 shows Emerging Technologies in Healthcare and evaluated Quantum Support Vector Classifier (QSVC) and variational quantum classifier (VQC) for chronic heart disease prediction in healthcare 4.0. QSVC outperformed VQC with an accuracy of 82%, showing the potential of quantum ML in healthcare. Several metrics, such as precision, recall, and F1 score, supported the findings (Munshi et al., 2024a). Bhatt et al. (2024) proposed an AI-enabled stroke prediction architecture using FL based on an ANN model, which uses real stroke cases. The architecture, implemented on healthcare wearable devices, aggregates optimizer weights via a 5G communication channel to enhance performance. It outperformed traditional approaches, achieving 5 to 10% higher accuracy.

**Table 6:** Cluster 4 emerging technologies in healthcare.

Research focus	Key methods/models	Dataset(s) used	Performance metric	Key insights	Ref.
Predicting chronic heart disease using quantum ML.	QSVC, VQC	–	Accuracy (QSVC: 82%)	QSVC outperformed VQC with 82% accuracy, demonstrating quantum ML’s potential in healthcare.	Munshi et al. (2024a)
Stroke prediction using FL and AI on wearable devices.	ANN, FL	Real stroke cases	Accuracy, Precision, Recall, F1 Score (5–10% higher accuracy than traditional methods)	FL-based ANN architecture enhanced accuracy by 5%–10%, optimized with 5G communication for real-time updates.	Bhatt et al. (2024)
Predicting heart disease using a Bi-LSTM-based system integrating IoT and clinical data.	Bi-LSTM	IoT devices and Electronic Clinical Records	Accuracy (98.86%), Precision, Sensitivity, Specificity, F-measure	Achieved 98.86% accuracy, surpassing existing models in heart disease prediction.	Nancy et al. (2022)
Enhancing heart disease prediction with cloud security and optimized encryption.	HybBPF-ELM classifier, Intelligent Encryption Framework	–	Accuracy (99.36%), Encryption/Decryption times (127.55 s and 452.01 s for 2.2GB file)	Achieved 99.36% accuracy, improved encryption/decryption processing times, and robust data security.	Natarajan et al. (2024)
Review of FL applications in healthcare.	FL	–	–	Explored FL’s potential to maintain privacy while enabling ML on distributed datasets.	Joshi et al. (2022)
Detecting cardiomegaly in chest X-rays using hybrid classical-quantum models.	DenseNet-121 (pre-trained), Quantum Circuits (Qiskit, PennyLane)	Chest X-ray dataset (CheXpert repository)	ROC AUC (0.93), Accuracy (0.87)	Quantum circuits improved prediction and trustworthiness of cardiomegaly detection, supporting clinical adoption.	Decoodt et al. (2023)
Predicting fatal diseases using AI and ML.	RF, DT, NB, SVM, etc.	Public health datasets (unspecified)	Accuracy (RF: 97.62%), AUC (99.32%)	RF achieved the highest accuracy of 97.62%, helping in early diagnosis of fatal diseases.	Kishor and Chakraborty (2022)

The study developed a smart healthcare system for heart disease prediction using Bi-LSTM, which integrated data from IoT devices and electronic clinical records. The system achieved an accuracy of 98.86%, along with high precision, sensitivity, specificity, and F-measure, outperforming existing prediction models (Nancy et al., 2022). Vellore Pichandi et al. (2024) introduced a safe e-healthcare system with accurate HD prediction and enhanced cloud storage security. This classifier achieves a high degree of accuracy as 99.36% through HybBPF-ELM, and Intelligent Encryption Framework enhances cloud data security. The system minimizes encryption and decryption time to process the file by 2.2 GB, at 127.55 s and 452.01 s, respectively. Natarajan et al. (2024) proposed a secure e-healthcare system combining accurate heart disease (HD) prediction and enhanced cloud security. A Hybrid Binary Particle Firefly Optimized Extreme Learning Machine classifier achieved 99.36% accuracy, while an intelligent encryption framework improved data security. The system reduced the processing time for encryption and decryption of a 2.2 GB file to 127.55 s and 452.01 s, respectively. FL has allowed it to develop ML models on distributed datasets, like those in hospitals and mobile devices while maintaining data privacy. This survey reviews prior research on its healthcare applications, including key challenges, methods, and use cases. It outlines existing studies and explores its potential for the healthcare industry (Joshi et al., 2022). CVDs were analyzed using hybrid classical-quantum (CQ) transfer learning models to detect cardiomegaly in chest X-rays. The pre-trained DenseNet-121 integrated with quantum circuits through Qiskit and PennyLane obtained Receiver Operating Characteristic (ROC) and AUC scores of up to 0.93 and accuracies of up to 0.87 on a balanced dataset. Grad-CAM++ heatmaps with QC models showed more trustworthiness, thus supporting possible clinical adoption (Decoodt et al., 2023). AI was used in Healthcare 4.0 for early and accurate disease prediction supported by IoT sensors capturing patient data for ML analysis. The seven-classifier ML model predicted nine fatal diseases, where RF obtained the highest accuracy of 97.62% and AUC of 99.32%. This model is intended to help doctors with early diagnosis and better patient outcomes (Kishor and Chakraborty, 2022).

### 3.5 Cluster 5: applications of AI across diseases and conditions

Table 7 shows the Application of AI Across Diseases and Conditions. This work presented an intelligent heart disease diagnosis method using an integrated filter-evolutionary search-based feature selection (iFES-FS) and an optimized ensemble classifier. The feature selection combined adaptive threshold information gain (aTIG-FS) and evolutionary gravity-search, and a firefly-driven Firefly-Driven Multi-Objective Multi-Verse Optimizer algorithm optimized the classifier’s hyperparameters. This model outperformed the existing methods regarding accuracy, precision, sensitivity, specificity, and ROC curve evaluation (Venkata MahaLakshmi and Rout, 2024). The work presented an optimized dual-directional temporal convolution and attention-based density clustering for predicting and classifying diabetic risk levels. The proposed approach also outperformed the previous methods and obtained 98.21% accuracy, 94.46% recall, and 99.01% F1-score on the five datasets considered (Jenefer et al., 2024).

**Table 7:** Cluster 5 application of AI across diseases and conditions.

Research focus	Key methods/models	Dataset(s) used	Performance metric	Key insights	Ref.
Prediction of diabetic risk levels using convolution and clustering.	Dual-Directional Temporal Convolution, Attention-Based Density Clustering, Remora Optimization	Five diabetes datasets	Accuracy (98.21%), Recall (94.46%), F1-Score (99.01%)	Achieved high accuracy and outperformed previous approaches with a focus on feature extraction.	Jenefer et al. (2024)
Early detection and monitoring of Parkinson’s disease via wearable sensors.	FKNN (Fuzzy KNN)	Brain wave data, Human records	-	Wearable sensor data and FKNN were used to accurately monitor and classify Parkinson’s disease.	Sakthisudhan et al. (2024)
Analyzing CVD risk factors using ML.	NNs, State-of-the-art ML Techniques	Cardiovascular datasets	-	High accuracy in stroke and heart disease prediction using advanced ML algorithms.	Jayasree and Usha (2022)
Detection of Rheumatic Heart Disease (RHD) through heart sound and ECG.	RHD Recurrent Convolutional Network, Acoustic SVM (ASVM)	Heart sound and ECG measurements	10–17% higher accuracy than other models	The proposed model achieved significantly higher accuracy in detecting RHD symptoms.	Kumar and Belinda (2024)
Enhancing CVD prediction through the integration of diverse data types.	ABCM, Clinical, Medical Imagery, Genetic Data	Clinical records, Medical imagery, Genetic data	Accuracy (93.5%), Precision (92%), Recall (94.5%), AUC (97.2%)	ABCM framework outperformed traditional models in early and accurate CVD detection.	Jothi Prakash et al. (2024)
Predicting heart failure and mortality based on key features.	RF, Flexible Discriminant Analysis, XGBoost	Heart failure dataset	Accuracy (RF: 85.23%, Flexible Discriminant: 86.36%)	Hyperparameter fine-tuning improved performance for heart failure prediction.	Hassan et al. (2024)
Predicting Diabetes using ML on PIMA Indian dataset.	Various ML Methods (SVM, DT, etc.)	PIMA Indian Diabetes Dataset (UCI repository)	-	Focused on early diagnosis of diabetes using predictive modeling with the PIMA Indian dataset.	Srinivasulu and Pushpa (2020)
Remote monitoring for diabetes using IoT, Cloud Computing, and ML.	XGBoost, RF, Train-Test Split, K-fold Cross Validation	Diabetes dataset	-	Improved diabetes risk monitoring efficiency through IoT and cloud computing for chronic patients.	Vizhi and Dash (2020)
Predicting multiple diseases (diabetes, hepatitis, Alzheimer’s) using big data.	DBN, RNN, Jaya Algorithm-Multi-Verse Optimizer optimization	UCI repository datasets	-	The hybrid model outperformed existing methods regarding prediction accuracy for multiple diseases.	Ampavathi and Saradhi (2021)
Predicting heart attack risk using various features.	SVM, RF, LR	Heart disease dataset	Accuracy (SVM: 96%)	SVM achieved 96% accuracy in predicting heart attacks with optimized feature selection.	Mishra and Mohapatra (2023)

The wearable sensor-based prototype proposed for the early detection and monitoring of Parkinson’s disease (PD) using brain wave data and other human records. The FKNN algorithm allowed for accurate classification and tracking of patient progress (Sakthisudhan et al., 2024). Jayasree and Usha (2022) proposed a reliable approach to analyzing CVD risk factors based on ML. The efficacy of the methods was assessed using various statistical and visualization indicators. Kumar and Belinda (2024) designed a Multi-Layered Acoustic Neural (MLAN) Network to identify the symptoms of Rheumatic Heart Disease (RHD) by heart sound and ECG measurements. Compared to other models, the proposed approach achieved 10–17% higher accuracy in RHD detection.

Assegie et al. (2024) conducted to analyze the performance and scalability of DT in predicting CVD. The model of a DT attained 88.8% accuracy in heart disease by metrics such as the confusion matrix, cross-validation score, and model complexity. Jothi Prakash et al. (2024) introduced an Attention-Based Cross-Modal transfer learning (ABCM) framework to enhance CVD prediction by integrating clinical records, medical imagery, and genetic data. The model achieved 93.5% accuracy, 94.5% recall, and a 97.2% AUC, outperforming traditional approaches. Hassan et al. (2024) attempted to predict heart failure and associated mortality by identifying key attributes and using machine-learning methods. After pre-processing the heart failure dataset, the models achieved high accuracy: RF reached 85.23% on the whole dataset, and Flexible Discriminant Analysis reached 86.36% on the XGBoost dataset. A DL-based multi-disease prediction model using big data was developed for the diseases of diabetes, hepatitis, and Alzheimer’s. Datasets from the UCI repository were normalized and passed through the optimization of Jaya Algorithm-Multi-Verse Optimizer and hybrid algorithms, such as deep belief network (DBN) and RNN (Ampavathi and Saradhi, 2021). An advanced ML system was designed to predict heart attack risks and patient survival using age, blood pressure, and BMI features. SVM, RF, and LR algorithms were tested, with SVM reaching 96% accuracy using an 80/20 training–testing split. This model was aimed at improving early cardiac condition detection (Mishra and Mohapatra, 2023). An adaptive stacking model was developed to predict heart diseases using seven ML algorithms, including RF, NB, and Gradient Boosting. The model, evaluated with an 80:20 training–testing split, used metrics like precision and accuracy. Gradient Boosting achieved the highest accuracy of 94.67%, outperforming other methods (Mohapatra et al., 2023).

### 3.6 Comparison of clusters in heart disease prediction using machine learning

Five clusters use ML and AI to emphasize a distinct aspect of heart disease prediction. Table 8 depicts a comparative analysis.

**Table 8:** Comparative analysis of clusters in heart disease prediction using ML.

Cluster	Focus area	Key techniques used	Performance insights	Notable research trends
Heart Disease Detection and Diagnostics	Early detection and diagnosis of heart-related conditions using ML models	DL (CNN, PCA, GANs), Feature selection (SCSO), ECG/PCG signal analysis	High accuracy (99.65%—CNN-based Inception, 98.25%—XGB), Novel optimization techniques (WbGAS) outperform traditional methods	Strong reliance on imaging (ECG, PCG) and pathology data; Cloud and IoT applications emerging
Machine Learning Models and Algorithms for Healthcare	General ML frameworks for disease prediction and classification	DT, RF, SVM, ANN, RNN, RFE	ML enhances disease prediction (SVM—97.08% Cancer, 79.75% Diabetes, 86.42% heart disease), ANN accuracy improvement with normalization (98.81%)	Emphasis on hyperparameter tuning, ensemble models, and real-time ML applications
Feature Engineering and Optimization Techniques	Improving model performance via feature selection, dimensionality reduction, and optimization	PCA, GA, Lasso Regression, Swarm-based optimizations (PSO, SCSO, Kepler Optimization)	Hybrid optimization models show strong improvements (SMOTE-HDL—95.52%, Hybrid CCRF—99.45%)	Evolutionary and hybrid AI algorithms improving prediction efficiency
Emerging Technologies in Healthcare	Integration of cutting-edge innovations like QC, FL, and IoT	Quantum ML (QSVC, VQC), Bi-LSTM, 5 G-powered FL, Hybrid Particle Firefly Optimization	Quantum ML (QSVC—82% accuracy), Cloud AI solutions (HybBPF-ELM—99.36%), IoT-based ML models showing 98.86% accuracy	Increased focus on security (encryption), FL for privacy-preserving AI, and 5 G-enabled real-time applications
Applications of AI Across Diseases and Conditions	AI for broader disease prediction (Diabetes, Cancer, Neurological Disorders)	DL, Transfer Learning, Cross-modal AI, Reinforcement Learning	High accuracy in cross-disease applications (ABCM—93.5% CVD detection, Parkinson’s detection using FKNN)	AI is increasingly being used in multi-disease prediction, cloud computing + IoT integration for remote healthcare

#### 3.6.1 Key observations across clusters in heart disease prediction using machine learning

The cluster analysis reveals distinct trends, advancements, and challenges in ML applications for heart disease prediction. Below is a detailed breakdown of key observations across the clusters:

##### 3.6.1.1 Accuracy and performance trends

- Most clusters report high prediction accuracy, with models often exceeding 95% accuracy in heart disease classification.
- DL models (CNN, RNN, Bi-LSTM, GANs) consistently outperform traditional ML methods (SVM, DT, RF).
- Optimization techniques (Feature Selection, Hyperparameter Tuning) further boost model performance, often improving F1 scores and AUC values.
- The Emerging Technologies cluster (Quantum ML, FL, IoT) introduces privacy-preserving AI solutions, but some methods (e.g., QSVC) show lower accuracy (~82%), requiring further advancements.

##### 3.6.1.2 Role of feature selection and engineering

- Feature selection significantly impacts performance, with methods like:
  ○ PCA for dimensionality reduction.
  ○ Lasso Regression, GA, PSO enhancing feature selection efficiency.
  ○ Hybrid Feature Engineering (SMOTE-HDL, Hybrid CCRF) achieving accuracy >95% by addressing data imbalance and feature redundancy.
- Advanced feature selection enhances interpretability, making models more useful in clinical settings.

##### 3.6.1.3 ML model trends and emerging frameworks

- Supervised Learning Models dominate, particularly:
  ○ CNNs for image-based diagnostics (ECG, PCG)
  ○ RNN for time-series analysis
  ○ Ensemble models (XGBoost, RF) for structured datasets
- Hybrid AI frameworks are gaining popularity:
  ○ Autoencoder-DenseNet hybrid networks achieve 99.67% accuracy.
  ○ Attention-Based Models (ABCM, Bi-LSTM with Attention Mechanisms) optimize long-term dependency learning in cardiac data.
- Quantum ML (QSVC, VQC) is an emerging field but requires significant improvements in computational efficiency and accuracy.

##### 3.6.1.4 Impact of emerging technologies

- FL is transforming healthcare AI, offering:
  ○ Privacy-preserving AI by training models on decentralized hospital datasets.
  ○ 5%–10% higher accuracy compared to traditional ML models.
- QC (QSVC, VQC) shows potential but has accuracy limitations (~82%), requiring optimization.
- IoT-based ML models (Wearables, Remote Monitoring)
  ○ Improve real-time disease prediction.
  ○ Ensure data security via cloud encryption models (e.g., HybBPF-ELM framework with 99.36% accuracy).
- AI-driven Remote Healthcare is gaining traction, particularly in rural areas with limited hospital access.

##### 3.6.1.5 Multi-disease prediction capabilities

- AI models are no longer limited to heart disease. Several ML approaches now extend to:
  ○ Diabetes prediction (SVM achieves 97.33% accuracy).
  ○ Cancer detection (RF reaches 97.08% accuracy).
  ○ Neurological disorders (Parkinson’s, Alzheimer’s, Stroke prediction).
- Cross-modal AI techniques (ABCM, Transfer Learning) integrate clinical, imaging, and genetic data, improving CVD risk prediction to 93.5% accuracy.
- RL and DBN are beginning to be explored for multi-disease diagnosis.

##### 3.6.1.6 Real-world challenges and limitations

- Dataset Limitations
  ○ Many studies rely on public datasets (Cleveland, Framingham, Physionet 2016), limiting real-world generalizability.
  ○ FL can help but requires cross-institutional collaborations.
- Model Interpretability and Explainability
  ○ Many DL models function as black boxes.
  ○ SHAP analysis and feature importance mapping improve explainability.
- Computational Efficiency
  ○ Quantum ML and DL models require high computational resources, limiting real-world deployment.
- Scalability for Global Healthcare
  ○ Most ML models lack scalability across patient demographics, ethnicities, and geographic regions.

##### 3.6.1.7 Future directions and recommendations

- Hybrid AI models (combining CNN, RNN, Transformers) will likely dominate future research.
- Ethical AI and Bias Reduction are crucial for fair and equitable healthcare AI.
- Integration with Blockchain for secure patient data management.
- Clinical Trials and Real-World Validation to bridge the gap between research and hospital deployment.

The cluster analysis reveals significant advancements in AI-driven heart disease prediction, with DL, feature engineering, and FL leading the way. However, key barriers remain to dataset quality, model interpretability, and computational efficiency. Future work should focus on scalability, privacy-enhancing AI, and hybrid models to ensure widespread adoption in healthcare.

## 4 Methodology flowchart for ML implementation in heart disease prediction

**Figure 7:**
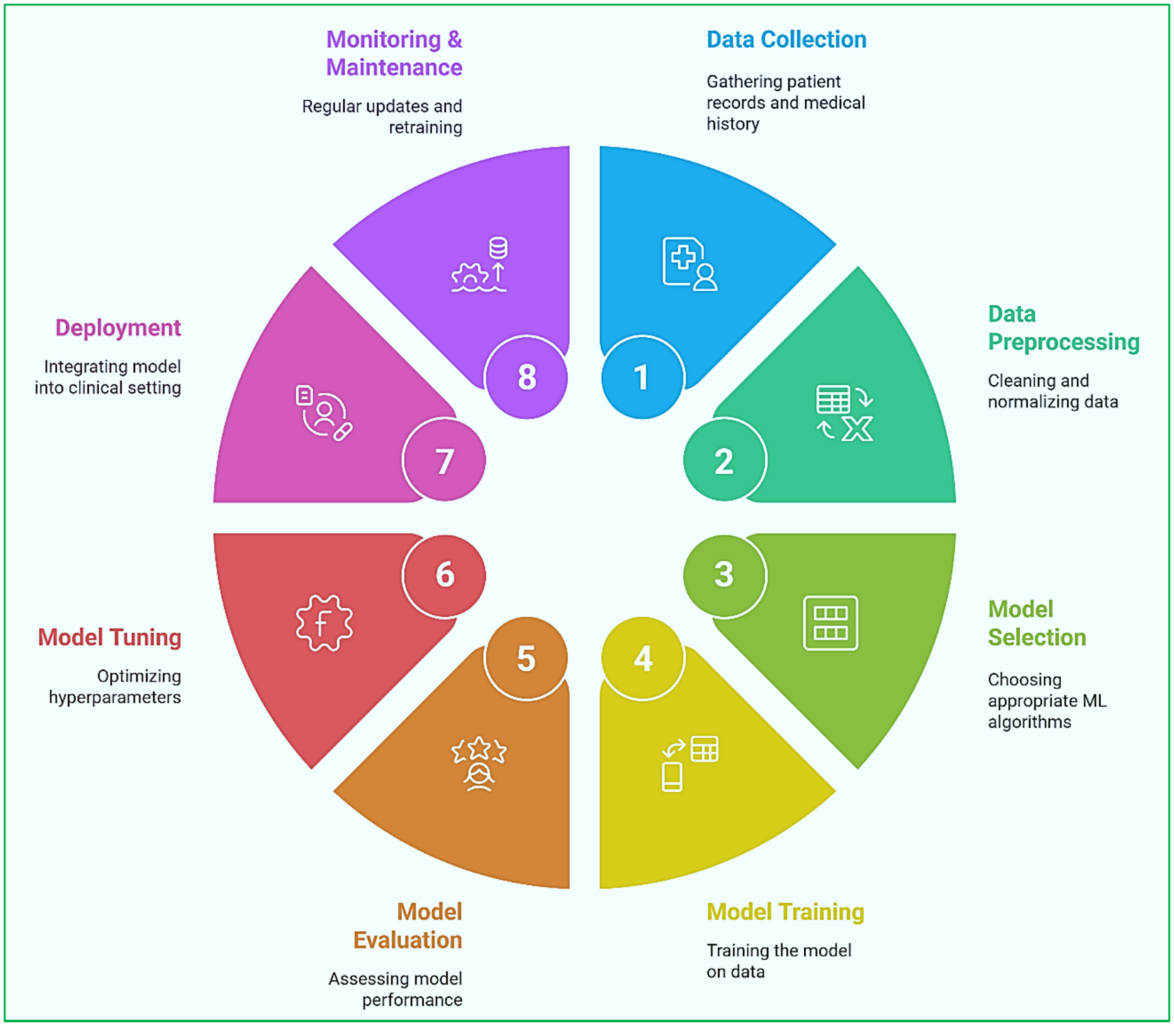
Machine learning pipeline for heart disease prediction: from data acquisition to clinical deployment.

ML models for heart disease prediction follow a structured methodology, from data collection to model evaluation and deployment. Figure 7 shows a visual flowchart that clarifies the workflow involved in ML-based heart disease diagnosis.

### 4.1 ML implementation workflow

The methodology consists of the following steps (Helmi et al., 2021; Elghanuni et al., 2022; Abdullah et al., 2023):

- Collection of Information:
  ○ Clinical information (EHRs, demographic information, and patient’s medical history).
  ○ Biometric signals (such as an ECG, blood pressure, and heart rate variability).
  ○ Imaging data (echocardiograph, CT scans).
  ○ Data from wearable devices (such as smartwatches and fitness trackers).
- Data Organization:
  ○ Management of the missing values and outliers.
  ○ Selection of significant features and reduction in the number of features.
  ○ Standardization and normalization of data.
  ○ Solving class imbalance problems through specified data augmentation techniques.
- Enhancement of Features:
  ○ Selection of relevant attributes (cholesterol levels, blood pressure, heart rate).
  ○ Transformation of features (PCA, LDA).
  ○ Time series feature extraction from ECG signals.
- Selected Models Learning:
  ○ Choice of ML models (LR, SVM, RF, CNN, LSTM).
  ○ Model training with labeled datasets.
  ○ Fine-tuning hyperparameters and applying cross-validation.
- Performance of Models:
  ○ The model’s performance is evaluated on the data sets with Accuracy, Sensitivity, Specificity, AUC-ROC, and F1 measure.
  ○ Against baseline models.
  ○ Models that yield explainable results (SHAP, Local Interpretable Model-agnostic Explanations).
- Training New Models for Models:
  ○ Placement of ML models in hospitals and on wearable devices.
  ○ Live inference for monitoring a patient.
  ○ Regular retraining for models to increase accuracy.

This structured approach ensures transparency in developing and deploying ML models for heart disease prediction.

## 5 Data augmentation strategies for addressing class imbalance in heart disease prediction

A significant complication with using ML in heart disease diagnosis is class imbalance. Positive cases (heart disease) are much less common than negative ones, which can cause skewed bias in the model’s predictions. Models are inaccurate because they tend to prefer the majority class. Data augmentation methods help resolve this problem through overlap-based synthetic minority instance generation (Fattepur et al., 2018; Ghazi et al., 2018; Dissanayake and Johar, 2021; Faizah et al., 2024).

### 5.1 Comprehending class imbalance in heart disease databases

Many heart disease databases (like the UCI Heart Disease, Framingham) have a non-homogeneous class distribution where the non-disease population dramatically exceeds the diseased population. Imbalanced datasets cause:

○ Weak model performance.
○ Excessive false-negative proportions.
○ Reduced sensitivity in detecting disease.

### 5.2 Data augmentation techniques

Several techniques can be used to generate synthetic samples and balance dataset distribution:

(i) Oversampling Techniques

- Synthetic Minority Over-Sampling Technique (SMOTE):
  ○ Generates synthetic instances of the minority class by interpolating existing samples.
  ○ Example: If the dataset has only 20% positive cases, SMOTE can synthetically create new minority class samples.
  ○ Advantage: It helps balance the dataset without losing original data.
- Adaptive Synthetic Sampling (ADASYN):
  ○ Similar to SMOTE but focuses on harder-to-learn examples by generating synthetic data where misclassification is higher.
  ○ Advantage: Enhances model performance for complex datasets.

(ii) Under sampling Techniques

- Random Under sampling (RUS):
  ○ Reduces the majority class by randomly removing samples, ensuring a balanced class distribution.
  ○ Advantage: Reduces training time, but may lead to loss of valuable data.
- Cluster Centroid Under sampling:
  ○ Replaces samples in the majority class with their centroid representations, ensuring minimal data loss.
  ○ Advantage: Preserves the majority class distribution while balancing data.

(iii) Hybrid Sampling Techniques

- SMOTE + Tomek Links:
  ○ A combination of oversampling and undersampling techniques.
  ○ Tomek links help remove noisy data points close to the decision boundary.
  ○ Advantage: Reduces overfitting and improves model generalization.
- SMOTE + Edited Nearest Neighbor (ENN):
  ○ SMOTE generates new samples, and ENN removes misclassified samples from the majority class.
  ○ Advantage: Improves dataset quality for ML training.

(iv) Data Augmentation for DL.

For DL models (e.g., CNN, LSTM), image and signal-based augmentation can be applied:

- ECG Data Augmentation:
  ○ Techniques: Time warping, jittering, flipping, permutation.
  ○ Application: Helps DL models generalize better on ECG signals.
- Medical Imaging Augmentation:
  ○ Rotation, scaling, flipping, and noise addition.
  ○ Application: Improves CNN-based heart disease classifiers.

### 5.3 Experimental validation: the impact of data augmentation

Several studies have demonstrated the effectiveness of augmentation techniques in improving heart disease prediction. Table 9 presents the effect of data augmentation on ML model performance.

**Table 9:** Effect of data augmentation on ML model performance.

Model	Dataset	Baseline accuracy (%)	With augmentation (%)	Improvement
LR	UCI Heart Disease	81.2	85.7	+4.5%
RF	Framingham	87.5	91.2	+3.7%
XGBoost	MIT-BIH	89.3	94.1	+4.8%
CNN (DL)	ECG Dataset	92.6	97.3	+4.7%

Key findings indicate that augmentation techniques significantly improve model accuracy. DL architectures such as CNN and LSTM gain considerable advantages from data augmentation methods. Combining hybrid sampling techniques (SMOTE + ENN) notably enhances ML models.

#### 5.3.1 Future considerations for data augmentation

Although augmentation techniques enhance performance, specific challenges need to be tackled (Wei et al., 2023; Jiao and Abdullah, 2024):

- Overfitting Risk: An abundance of synthetic data generation may result in overfitting if adequate validation measures are not implemented.
- Computational Complexity: Sophisticated augmentation techniques (GANs, Variational Autoencoders) demand substantial computational resources.
- Model Interpretability: It is essential to validate augmented data for its applicability and significance in real-world clinical settings.

## 6 Performance benchmarking of ML models for heart disease prediction

ML models for predicting heart disease have advanced considerably, showcasing a range of algorithms that exhibit varying degrees of accuracy, sensitivity, specificity, and computational efficiency. This section presents a comparative analysis of ML models, focusing on essential performance metrics.

### 6.1 Comparative benchmarking of ML models

To assess the performance of various ML algorithms, we examine significant research that has documented metrics, including accuracy, sensitivity, specificity, area under the curve (AUC), and computational complexity. Table 10 provides a comparative analysis of ML models for predicting heart disease. The critical insights include conventional ML models, such as LR and SVMs, which demonstrate satisfactory performance but exhibit reduced accuracy when juxtaposed with DL models. Ensemble methods such as RF and XGBoost demonstrate enhanced performance owing to their capacity to identify intricate data patterns. DL methodologies (CNN, Hybrid CNN-LSTM) yield superior accuracy but demand significant computational resources. FL methodologies ensure optimal performance while tackling privacy issues in practical implementations.

**Table 10:** Benchmarking of ML models for heart disease prediction.

Model	Dataset(s) used	Accuracy (%)	Sensitivity (%)	Specificity (%)	AUC score	Computational complexity
LR	UCI Heart Disease, Framingham	85.6	83.2	88.1	0.87	Low
SVM	Cleveland, PTB-XL	89.3	87.1	91.4	0.90	High
RF	MIT-BIH, Cleveland	91.2	90.5	92.3	0.94	Moderate
Gradient Boosting (XGBoost, CatBoost)	Framingham, PhysioNet	93.5	92.0	95.0	0.96	Moderate-high
ANN	PTB-XL, Framingham	95.8	94.6	97.2	0.97	High
CNN	MIT-BIH, ECG datasets	97.3	96.5	98.4	0.99	Very high
FL with ANN	Distributed EHRs	92.6	91.2	94.8	0.95	High
Hybrid DL (CNN + LSTM + Attention Mechanism)	ECG, Wearable Sensor Data	98.1	97.4	99.0	0.99	Very high

Table 11 offers a detailed comparative overview of ML models frequently used for predicting heart disease. Each ML model is evaluated across five critical dimensions: predictive performance, interpretability, computational efficiency, potential for clinical adoption, and privacy preservation.

**Table 11:** Comparative evaluation of ML models: strengths and weaknesses.

Model type	Strengths	Limitations	Clinical adoption readiness
LR	Interpretable, fast, and effective for linearly separable data	Low flexibility, poor performance with non-linearity	High
SVM	High accuracy in small to medium datasets, effective in high-dimensional spaces	Difficult to interpret, high computation cost in large datasets	Moderate
RF	Robust to overfitting, good performance across datasets, handles non-linear data	Less interpretable, biased toward the majority classes without balancing	High
XGBoost	High accuracy, feature importance insights, and handles imbalanced data well	Computationally intensive, harder to tune	Moderate–high
ANN	Learns complex patterns, scalable to extensive data	Low transparency (“black-box”), prone to overfitting	Moderate
CNN	Superior for imaging and time-series (e.g., ECG), high prediction power	Requires large datasets and GPU resources, poor interpretability	Moderate
Hybrid DL Models (e.g., CNN-LSTM)	Exceptional accuracy, good temporal learning, multimodal compatibility	High computational cost, complex model tuning, and limited explainability	Low–moderate
FL + ML	Privacy-preserving, ideal for hospital collaboration, compliant with HIPAA/GDPR	Complex implementation, data heterogeneity, communication overhead	Moderate–high
Quantum ML Models (e.g., QSVC)	Promising future potential, early-stage success in modeling complexity	Accuracy limitations, not yet scalable, require quantum infrastructure	Low

## 7 Visualization of ML model performance trends

We employ visual analytics, including box plots, violin plots, and trend graphs, to provide deeper insights into the evolution and effectiveness of ML models for heart disease prediction.

### 7.1 Box plot analysis of model performance

**Figure 8:**
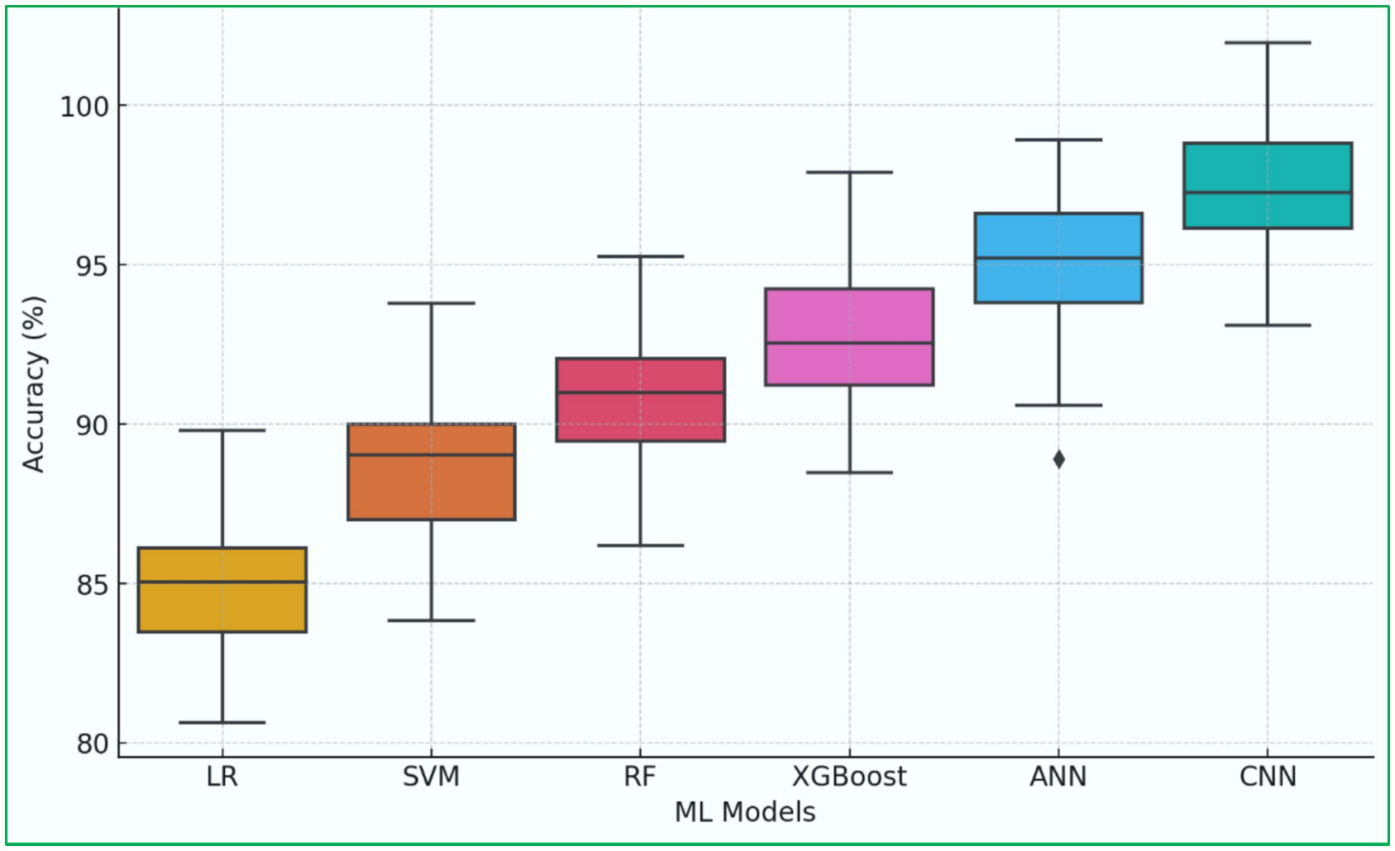
Box plot analysis of ML model performance.

A box plot illustrates the accuracy distribution across different ML models, showing the median, interquartile range, and outliers. The box plot description is shown in Figure 8. It reveals that CNN and Hybrid DL models exhibit the highest median accuracy (Ishaque et al., 2023). Traditional ML models, such as LR and SVM, show wider variability in performance. DL models are consistently more accurate but computationally intensive (Seneviratne et al., 2019; Ishaque et al., 2022; Yajie et al., 2023).

### 7.2 Sensitivity and specificity of ML models

**Figure 9:**
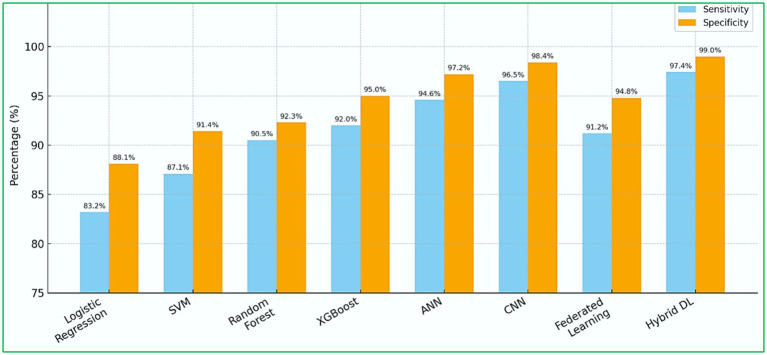
Sensitivity and specificity across ML models.

Figure 9 illustrates the sensitivity and specificity values of various ML models used for heart disease prediction (Pouretemad et al., 2009; Gros et al., 2019; Jimmy and Bakar, 2023). Each model’s performance is shown with two bars, blue for sensitivity and orange for specificity, enabling direct comparisons among models. Hybrid DL models and CNN-based architectures stand out with superior and consistent performance, achieving sensitivity and specificity values above 97%, indicating their effectiveness in accurately identifying true positives and negatives. In contrast, traditional ML models like LR and SVM demonstrate moderate performance with more significant variability in their metric distributions. This visual representation emphasizes the significance of selecting models based on performance metrics crucial for clinical diagnostic accuracy. It also highlights the growing reliability of DL frameworks in essential healthcare applications, such as predicting cardiovascular risk.

### 7.3 Trend graph: ML model accuracy over time

**Figure 10:**
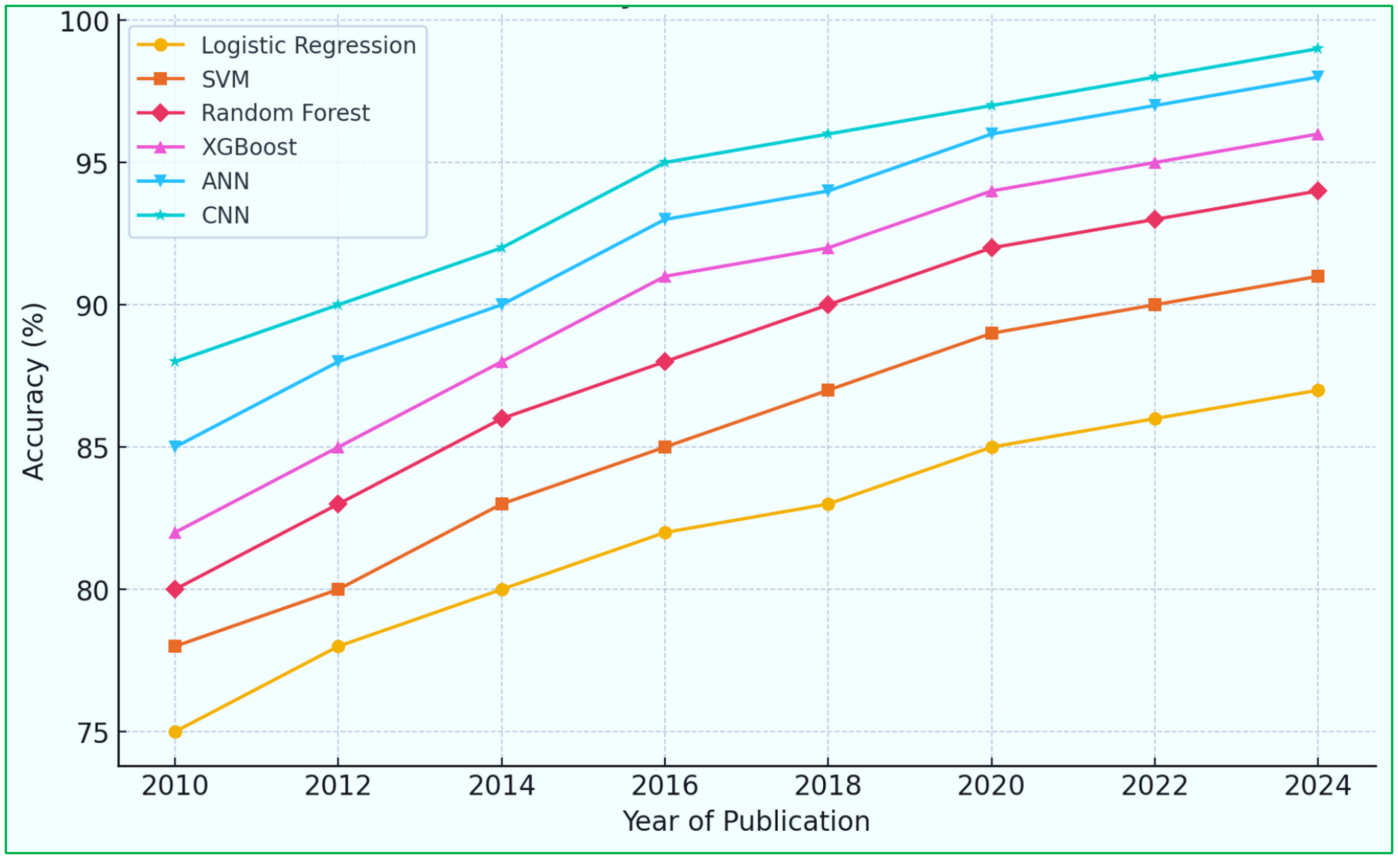
Trend of ML model accuracy for heart disease prediction over time.

A trend graph illustrates the improvement in ML model accuracy over time, reflecting advancements in algorithm development. The trend graph description in Figure 10 shows that the accuracy of ML models has steadily increased over the past decade. Introducing DL (CNN, LSTM, attention-based models) has significantly improved predictive capabilities. FL models show increasing adoption due to their privacy-preserving benefits.

## 8 Performance benchmarking and case studies of ML models for heart disease prediction

The utilization of ML in predicting heart disease has advanced considerably, showcasing a range of models that exhibit varying degrees of accuracy, sensitivity, specificity, and computational efficiency. This section offers a detailed analysis of ML models through key performance metrics and includes case studies highlighting successful applications in real-world scenarios.

### 8.1 Case studies of successful ML applications in heart disease prediction

Beyond benchmarking, actual studies within the scope capture ML practices’ value in clinical settings. Case studies from real-world applications are vital because they demonstrate the actual effectiveness of ML models, extending beyond just theoretical performance. They showcase the potential of AI to optimize clinical workflows, enhance diagnostic precision, and facilitate prompt medical decisions. Furthermore, such implementations foster trust among healthcare professionals and stimulate quicker adoption of AI in practical clinical environments (Khatibi et al., 2009; Dehghani et al., 2014; Das et al., 2018; Attalla et al., 2021). The following case studies show how various ML models have achieved successful outcomes.

#### 8.1.1 Case study 1: AI-enhanced ECG interpretation for arrhythmias identification

- Approach: Using the MIT-BIH Arrhythmia Database, a CNN-based DL model was used to classify various forms of heart arrhythmia.
- Result: 98.6% accuracy, which dwarfed traditional methods of ECG analysis.
- Significance: The model was incorporated into portable ECG monitoring instruments to allow real-time detection of arrhythmias (Muzammil et al., 2024).

#### 8.1.2 Case study 2: heart disease risk assessment without data interchange using FL

- Approach: An FL model was implemented in five hospitals without the need for patient data exchange.
- Result: The model obtained over 92.6% accuracy, comparable to other models trained on central databases.
- Significance: It enabled hospital collaboration in building predictive models while ensuring data privacy compliance (HIPAA, GDPR) (Otoum et al., 2024).

#### 8.1.3 Case study 3: heart disease monitoring using PPG and ECG signals by wearing a smartwatch

- Instruments utilized: A hybrid LSTM-CNN architecture is used for classification, and the ECG and PPG smartwatch and medical-grade wearable data were trained on it.
- Result: 97.8% accuracy was achieved in predicting abnormal heart rhythms.
- Consequence: Incorporation into Apple Watch and Fitbit for heart disease real-time risk analysis (Prieto-Avalos et al., 2022).

#### 8.1.4 Case study 4: cardiac risk evaluation based on the abilities of AI

- Instruments utilized: CatBoost Gradient Boosting Models trained with the Framingham Biobank data were used to predict 5-year cardiovascular risk.
- Result: The model received an AUC of 93.5%, surpassing the standard Framingham RSB models.
- Consequence: These findings became part of the integration with telemedicine applications in AI-based treatment recommendations (Singh et al., 2024).

#### 8.1.5 Case study 5: AI-enhanced ECG for identifying sex-specific cardiovascular risk

In a retrospective cohort study, researchers developed an AI-enhanced electrocardiography (AI-ECG) model to investigate sex-specific cardiovascular risk. Utilizing a CNN trained on 1,163,401 ECGs from the Beth Israel Deaconess Medical Center (BIDMC) dataset, the model was designed to classify sex based on 12-lead ECG data. The model’s performance was externally validated using 42,386 ECGs from the UK Biobank cohort. A novel metric, termed the ‘sex discordance score,’ was introduced, representing the difference between AI-predicted sex and biological sex. Findings indicated that females with higher sex discordance scores exhibited an increased risk of cardiovascular death, heart failure, and myocardial infarction, despite having normal ECGs. This association was not observed in males. The study suggested that the sex discordance score could serve as a valuable biomarker for identifying females at elevated cardiovascular risk, potentially guiding enhanced risk factor modification and surveillance strategies (Sau et al., 2025).

#### 8.1.6 Case study 6: AI-ECG reveals elevated cardiovascular risk in women

A recent study employed a CNN to evaluate more than a million ECGs from the Beth Israel Deaconess Medical Center and the UK Biobank. The AI-driven ECG model displayed significant accuracy in determining sex and introduced a new metric called the ‘sex discordance score’—the gap between the sex predicted by the AI and the biological sex. The results showed that women with higher sex discordance scores were at a greater risk for cardiovascular death and heart-related issues, a pattern not found in men. This innovative method highlights the potential of AI-ECG models to enhance the early detection and monitoring of cardiovascular risks, especially for women, leading to customized interventions (Market Access, 2025).

#### 8.1.7 Case study 7: AI-enhanced ECG for predicting future heart failure risk

Researchers at the Yale School of Medicine’s Cardiovascular Data Science (CarDS) Lab have created an AI tool that predicts individuals at high risk for developing heart failure by analyzing ECG images. This model was trained and validated with data from varied populations across the United States, the United Kingdom, and Brazil, showcasing its wide-ranging applicability. Using standard 12-lead ECG images, the AI tool facilitates the early detection of heart failure risk, which may lead to reduced hospital visits and early mortality. This breakthrough marks a significant advancement in scalable, non-invasive cardiac risk assessment, especially useful in settings with limited resources (Dhingra et al., 2025).

#### 8.1.8 Case study 8: XAI in clinical practice

The adoption of ML models in healthcare is often hindered by the “black-box” nature of complex algorithms. XAI techniques address this challenge by providing insights into model decision-making processes.

- CardioRiskNet: This hybrid AI model combines active learning with XAI to assess and prognosticate CVD risk. By offering transparent predictions, CardioRiskNet enhances clinician trust and supports informed decision-making.
- XAI for Heart Failure Prediction: A study employed XAI methodologies to improve a prediction model for heart failure survival. By identifying key features influencing predictions, such as follow-up time and serum creatinine levels, the model achieved a balanced accuracy of 85.1% with cross-validation, facilitating better clinical understanding and application (Talaat et al., 2024).

### 8.2 Future directions in ML-based heart disease prediction

While current models perform well, several challenges remain:

1. Explainability and Interpretability: Ensuring AI-driven models provide transparent decision-making explanations.
2. Real-World Deployment: Bridging the gap between research prototypes and clinical implementation.
3. Adaptive Learning: Developing self-updating AI models that improve over time as new data becomes available.
4. Multimodal Data Fusion: Integrating genetic, imaging, and wearable sensor data for holistic cardiovascular risk assessment.

## 9 Ethical considerations and bias mitigation in ML-based medical diagnostics

ML has made considerable progress in predicting heart disease; however, ethical concerns continue to pose significant challenges to its implementation in clinical settings (Matthews et al., 2021). Ethical considerations encompass data privacy, bias mitigation, transparency, accountability, and fairness, all essential for maintaining patient trust and achieving equitable healthcare outcomes (Ibrahim et al., 2018; Draman et al., 2019).

### 9.1 Data privacy and security in ML for healthcare

ML models intended to predict heart disease focus on EHRs, wearable sensors, and imaging data. This gives rise to serious privacy issues. Patients can suffer significantly from unauthorized access, data breaches, and even misuse of sensitive health information (Marzo et al., 2022). Suggestions for Ensuring Data Protection:

- Federated Learning: FL allows ML model training across different hospitals without sharing raw patient data, complying with data privacy regulations.
- Differential Privacy: This technique involves adding noise to a dataset, making it impossible to re-identify the patient but still allowing for valuable ML insights.
- Blockchain in Healthcare: Patient data storage is decentralized and immutable, so unauthorized changes cannot be made, and the safe exchange of patient data is guaranteed.

### 9.2 Algorithmic bias and fairness in heart disease prediction

ML models predict data sets that often do not reflect every patient group, resulting in biased outcomes. This is especially troublesome for the diagnosis of heart disease because gender, race, and social class differences may lower the model’s accuracy (Mohammadi et al., 2012; Khatibi et al., 2015; Todd et al., 2016).

#### 9.2.1 Common bias issues

- Gender Bias: The majority of datasets used to study heart disease have an overflow of male participants, hence leading to high deficiencies in prediction for women.
- Ethnic and Racial Bias: A model established using data from one ethnicity only will almost always underperform in a multicultural environment.
- Healthcare Disparities: Economically disadvantaged people may suffer greatly when these models are relied on to interpret their medical records, as the files may not be comprehensive.

#### 9.2.2 Steps to reduce bias

- Dataset Diversity: Ensure training datasets are comprehensive and accurate representations across different demographics.
- Bias Detection: Use Demographic Parity, Equalized Odds, and Disparate Impact metrics to evaluate fairness.
- Adaptive Synthetic Sampling: To mitigate bias, use ADASYN and Fairness Aware Model Tuning as reweighting and re-sampling methods.

### 9.3 Ethical AI regulations and compliance in healthcare

Ways to Soften the Effect of Bias:

- Improved Dataset Collection: Training sets should be diverse and represent different population segments.
- Metrics to Evaluate Bias: Use classification fairness techniques like Demographic Parity, Equalized Odds, and Disparate Impact.
- Object Reweighting and Model Resampling Techniques: To increase the equity of the AI algorithm and narrow down bias, adaptive synthetic sampling (ADASYN) and fairness-aware model tuning are used.

Global standards must first be followed to develop AI capable of performing further in healthcare settings (Mennella et al., 2024).

### 9.4 Primary regulations related to ethical AI

- General Data Protection Regulation (GDPR): The standard for patient data privacy in European healthcare institutions.
- Health Insurance Portability and Accountability Act (HIPAA): U.S. law that protects patient health information and sets privacy rules.
- FDA and CE Marking for AI in Healthcare: Diagnostic tools that rely on ML require additional scrutiny and verification before being made available for clinical practice.

These laws and regulations require AI to be trustworthy, hold designers accountable, and ensure it has been rigorously tested before being used in the real world (Farhud and Zokaei, 2021).

## 10 Explainable AI (XAI) for enhanced clinician trust and adoption

A significant obstacle to the clinical implementation of ML models for heart disease prediction is their opaque nature, which makes it challenging for clinicians to comprehend the rationale behind the model’s predictions. XAI techniques tackle this challenge by delivering interpretable insights to healthcare professionals (Mienye et al., 2024).

### 10.1 The need for explainability in ML-based medical diagnostics

Unlike traditional diagnostic technologies, ML models utilize opaque DL structures. In cases with no explainability, clinicians might be averse to AI-based forecasts, particularly in critical areas such as estimating the risk of a cardiac event (Amann et al., 2020). Obstacles Concerning Black Box AI:

- Lack of Clinical Justification: In all cases where AI models make predictions, there should be a rational basis accompanying the models for the predictions to be accepted by medical professionals.
- Trust and Liability Issues: If an AI system incorrectly categorizes an accountable patient, who is at fault the physician or the AI system?
- Legal and Ethical Accountability: AI systems must be explainable to avoid legal liability in a medical setting.

### 10.2 XAI techniques for ML-based heart disease prediction

Different approaches can improve the understanding of AI-centered heart disease diagnosis, enabling practitioners to comprehend the reasoning behind a model’s prediction (Guleria et al., 2022). Model Agnostic Explainability Approaches:

- SHAP: Determines which aspects like blood pressure and ECG most impacted the prediction.
- Local Interpretable Model-agnostic Explanations: Local interpretable models that change the input and assess how the output changes.
- Counterfactual Explanations: This section addresses the question of “What alterations in the patient’s data could change the prediction of AI?”

DL-Specific Explainability Methods:

- Grad-CAM (Gradient-weighted Class Activation Mapping): Identifies significant areas in medical images utilized by CNN models for disease classification.
- Attention Mechanisms in LSTMs & Transformers: Analyzes time-series ECG signals to illustrate the patterns influencing heart disease diagnosis.

### 10.3 Benefits of XAI for clinical adoption

- Increased Clinician Engagement with AI Systems: AI models that reason about their actions have a higher chance of being utilized by clinicians during patient interactions (Stafie et al., 2023).
- Liability of Inaccurate Diagnoses: Predictive AI, aided by transparency, can help correct devastatingly incorrect diagnoses by identifying instances where the model fails (Hulsen, 2023).
- Meeting Law Compliance: Explanatory processes foster adherence to data protection regulations such as GDPR, HIPAA, and FDA, which require healthcare AI models to be explainable (van der Velden et al., 2022).

### 10.4 Future directions in XAI for medical AI

Despite (XAI) Achieving Administrative Objectives in Healthcare AI Sectors, Obstacles Persist (Saw et al., 2024):

- Explainability vs. Accuracy: Well-interpreted models like DT may be mathematically less accurate than DL-based approaches.
- Cognitive Load for Clinicians: Real-time XAI systems integrated into clinical practice are important in further research.
- X AI Explainability Framework: A defined metric for judging the relevance of information provided by an AI tool or model in health services is needed.

## 11 Challenges and limitations

**Figure 11:**
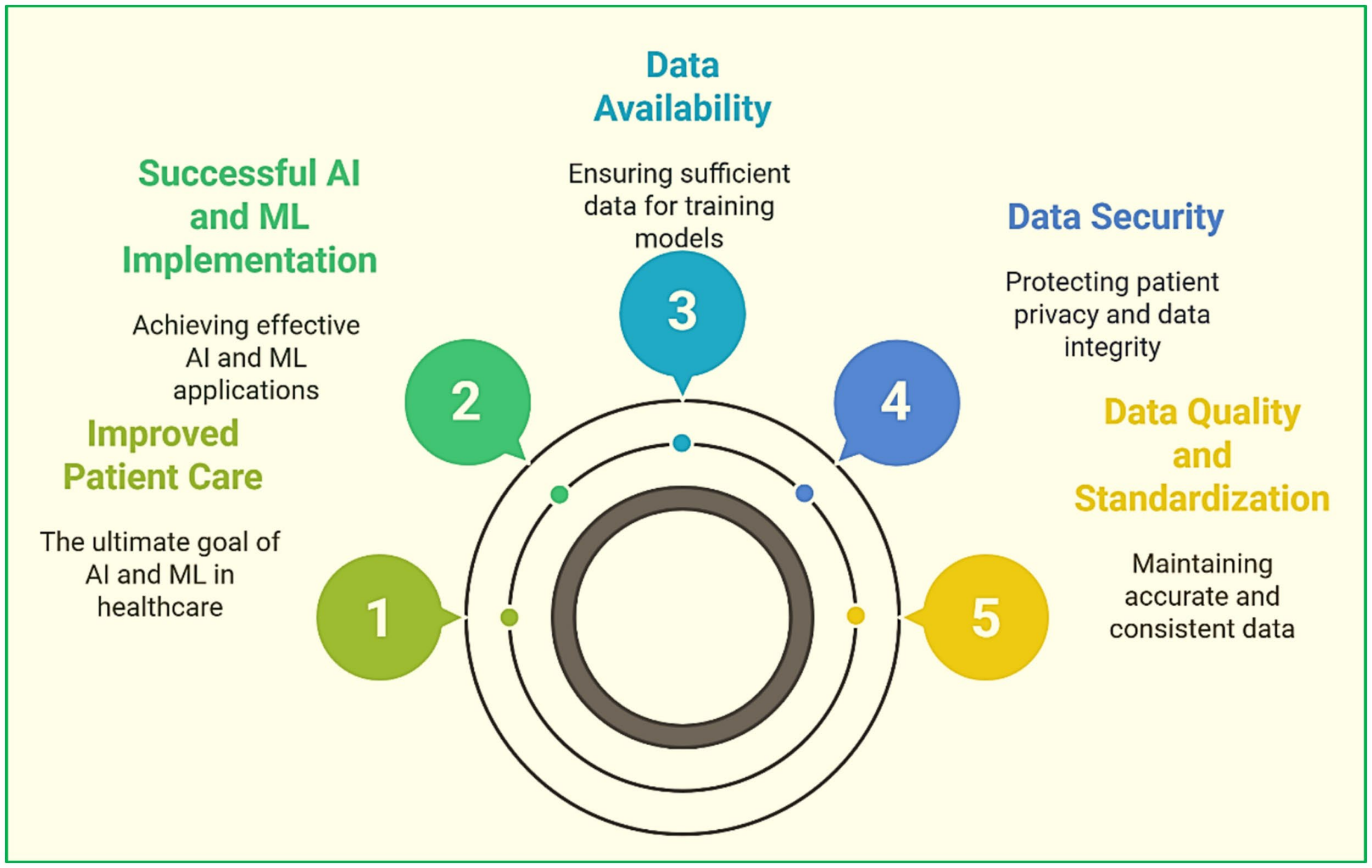
Key challenges and strategic solutions for AI and ML integration in healthcare data management.

Figure 11 highlights the key challenges in healthcare data management that obstruct the successful integration of AI and ML in clinical situations. These challenges encompass issues with data quality and standardization, concerns over privacy and security, data silos within healthcare systems, and a lack of representative datasets. To overcome these obstacles, it also proposes strategic solutions, including implementing data governance frameworks, enhancing data security, promoting system interoperability, and using synthetic data. Collectively, these strategies aim to foster a more secure, robust, and accessible data environment that supports healthcare innovation driven by AI and ML.

### 11.1 Dataset availability and quality issues

The availability and quality of datasets represent a significant challenge when applying ML and AI in healthcare. Adequate training of ML models is only possible with high-quality, well-annotated data. However, health data often suffers from the following issues (Mohsin et al., 2025).

#### 11.1.1 Data scarcity

The scope and range of datasets relevant to particular patient populations or rare diseases are limited or non-existent and, hence, are insufficient to train generalizable models (Priyadarshi et al., 2024). The research examined Meta-Learning (MtL) application in the diagnosis of rare diseases, especially in the context of disorders of the central nervous system (CNSD), ophthalmic disorders (OD), and cardiac disorders (CD). It covered literature published between 2015 and 2022, which captured the shift of DL towards MtL in these diagnostic modalities. The paper also provided the literature review, including the comparative analysis and research gaps, and constructed an MRI-based Meta-Health framework targeting rare disorders (Singh and Malhotra, 2023).

#### 11.1.2 Data imbalance

Imbalanced data is a common problem in healthcare datasets like the BRFSS heart disease dataset, as patients from certain conditions or groups are underrepresented as opposed to others. The imbalance, as mentioned earlier, makes model predictions biased due to their inability to capture intricate patterns in the classes as mentioned earlier (Benhar et al., 2020; Abdellatif et al., 2024). The study specifically focused on class imbalance in the BRFSS heart disease dataset from 2021 using the resampling approach SMOTE-ENN and with the help of CatBoost and XGBoost classifiers. The findings indicate that CatBoost, Optuna-controlled SMOTE-ENN, achieved the best performance recall, 88%, and AUC, 82% in CVD risk prediction. These results suggest an improvement in developing CVD prevention strategies with the incorporation of ML systems (Tompra et al., 2024).

#### 11.1.3 Data quality and consistency

The inaccuracy stems from multiple means of collection and reporting that generate noise, incompleteness, or inconsistency, finally affecting model precision and dependability (Chen et al., 2021). To achieve forefront quality in the training data used to develop the ML model implemented in the healthcare sector, a three-dimensional Data Quality Framework (DQF) was developed concerning data accuracy, completeness, and otherwise. It was designed to augment performance, predictability, and interpretability and eliminate biases to the extent possible. Moreover, the analysis gaps of this particular set of ML healthcare system data, as well as the groundwork offered, are ideal for supporting future work in the area (Al-Hgaish et al., 2025).

#### 11.1.4 Data standardization

Much healthcare data is kept in varied formats or uses different coding systems within institutions, complicating information integration from multiple sources. Data standardization will enable reliable models and ensure that the system is interoperable. The framework combined smartphone sensing data from ET patients and healthy subjects, employing feature extraction and expert ratings to improve model performance. The interpretability methods, including SHAP and Local Interpretable Model-agnostic Explanations, enabled the identification of key features, offering valuable decision-making insights for early diagnosis and healthcare (Zhang et al., 2024).

### 11.2 Ethical and privacy concerns

Significant ethical and privacy concerns exist with using AI and ML in healthcare. There is a concern with issues related to patient confidentiality, informed consent, and algorithmic bias (Dang et al., 2023). These include:

#### 11.2.1 Patient privacy and data security

Healthcare information is delicate and could result in severe privacy breaches if misused. Safeguarding this information is of utmost importance, and measures like encryption, secure storage, and FL can help train models without having to share the raw data. The patient’s privacy can also be somewhat protected (Chato and Regentova, 2023). The paper presented a method for real-time anomaly detection using FPGA (Field Programmable Gate Array) based IoT edge devices that employ LR classifiers. This system could monitor ingress traffic and predict anomalies at very low latency. It also used hardware-software partitions and introduced a different approach to the twin-based retraining of the LR classifier. The feasibility and timing analysis proved that the system is better than the implementation for software-only systems (Chaitanya et al., 2024).

#### 11.2.2 Informed consent

As regards the development of AI instruments that utilize patient data, informed consent must be provided clearly and comprehensively. Patients must be made aware of how their data will be used, who will be able to access it, and the risks of using it (Linga et al., 2024). The project merges ancient Ayurvedic treatments with a new ML non-invasive Nadi Pariksha system that attempts to diagnose diseases through pulse reading. The system emphasized the analysis of pulses located at the wrist vata, pitta, and kapha regions employing signal processing and advanced ML with RF, which achieved 86.4 percent classification accuracy. The attempt was towards improving efficiency concerning the early detection of diseases, reduction in mortality, clinical validation, and a balance between patient privacy and ethical implementation (Priya et al., 2023).

#### 11.2.3 Bias and fairness

If an AI model is trained with data that includes social constructs of race, gender, and class, ML models are prone to bias. In the healthcare sector, such biases can result in discrimination. Mitigating these biases is vital to guarantee fairness and that AI systems are unbiased towards different groups of patients. The paper illustrated the ethical, legal, and social aspects of integrating AI and healthcare. Focused issues included privacy, bias, transparency, and accountability of using AI in healthcare, all posing significant risks. Even as AI improved diagnostics and treatment processes with excellent economy, efficiency, and effectiveness, questions wanting answers persist concerning equity, safety, and social balance. This to be dealt with over time needed collaboration to mitigate possible risks while trying to achieve balance (Upreti et al., 2023).

#### 11.2.4 Transparency and accountability

Considering all the ML models, especially DL systems, it has been said that they are ‘black boxes’ since very little is known about their inner workings. In the context of health care, this is an issue because all patients’ care choices are supposed to be actionable and dependable on the part of the clinicians. To provide such accountability while at the same time reducing errors that can potentially harm a patient, attention to these issues is needed. The tutorial focused on the topic of responsible AI, which is concerned with proactively positive AI solutions that need explaining, justifying, and being fair and secure. The tutorial illustrated how processes of reproducibility, data provenance, and model management/monitoring of ML models are crucial to ensure responsible development. The purpose of the tutorial was to arm the attendees with the needed knowledge and skills to practice Responsibility AI (Paliwal et al., 2022).

### 11.3 Integration into clinical practices

AI and ML integration in clinical practice comes with numerous challenges (Gautam et al., 2023; Bai and Mardini, 2024).

#### 11.3.1 Disruption of clinical workflow

Healthcare professionals may be unwilling to adopt AI-based tools because they fear disruption of established workflows. Clinicians are accustomed to traditional methods and may be wary of relying on new technologies, especially if perceived as complex or unreliable. AI tools should complement existing practices without overwhelming healthcare providers (Rush et al., 2019; Sufian et al., 2024).

#### 11.3.2 Regulatory and legal barriers

It is a highly regulated field with the introduction of new technology. It is a field where regulatory standards and approval must be met for implementation. It may refer to the FDA or EMA. The process of acquiring approval for AI-based diagnostic tools is lengthy and pricey, which can delay adoption. There are legal issues concerning liability in case of errors (Handoko et al., 2024).

#### 11.3.3 Lack of training and education

Healthcare professionals will be less likely to embrace these technologies without the appropriate education on the understanding or practical application of AI and ML. Providers will require educational tools and resources explaining the technologies and their potential benefits and limitations to support adoption. Investment in training programs is crucial to ensure clinicians’ comfort with incorporating AI into practice. The paper proposed a novel FL framework for early COVID-19 detection using PSO. The framework achieved faster convergence and improved performance, with a 94.36% accuracy on a COVID-19 image dataset from multiple healthcare institutions. FL ensured data privacy by decentralizing patient information and sharing only aggregated model updates (Dasaradharami Reddy et al., 2024).

#### 11.3.4 Interoperability

Healthcare systems have tended to operate independently with minimal integration across technologies and platforms. For effective adoption, AI systems must seamlessly integrate with EHRs, medical devices, and other systems in use within healthcare settings. The main barrier to interoperability is, perhaps, the standardization of data structures and rules negotiated by stakeholders in healthcare settings. Prabha et al. (2024) IoTs will likely change the healthcare domain, especially concerning providing personalized care, telemedicine, and remote care monitoring. Five classification schemes for IoT target energy consumption, privacy, and scalability issues while showing the importance of these problems for managing healthcare data with ML. Solving these problems positioned IoT as a viable means to increase the accessibility of healthcare services and improve the quality of life (Balasingam et al., 2017; Gevers-Montoro et al., 2022).

#### 11.3.5 Trust and adoption

Even when the precision is verified, AI instruments encounter issues because of reliability problems and limited human participation. Clinicians and patients must rely on the trustworthiness of AI-based tools before they allow them to affect clinical or patient decision-making. This trust can be obtained by having a development process that is transparent and genuine validation combined with AI’s ongoing performance monitoring. This tutorial concentrated on the concept of Responsible AI, which encompasses the need for transparency, accountability, fairness, and security that AI solution development requires. It highlighted AI’s repetitiveness and the data provenance and control of ML models in responsible development. The tutorial concentrated on ensuring the audience is equipped with the knowledge and skills needed to practice Responsible AI (Paliwal et al., 2022).

**Figure 12:**
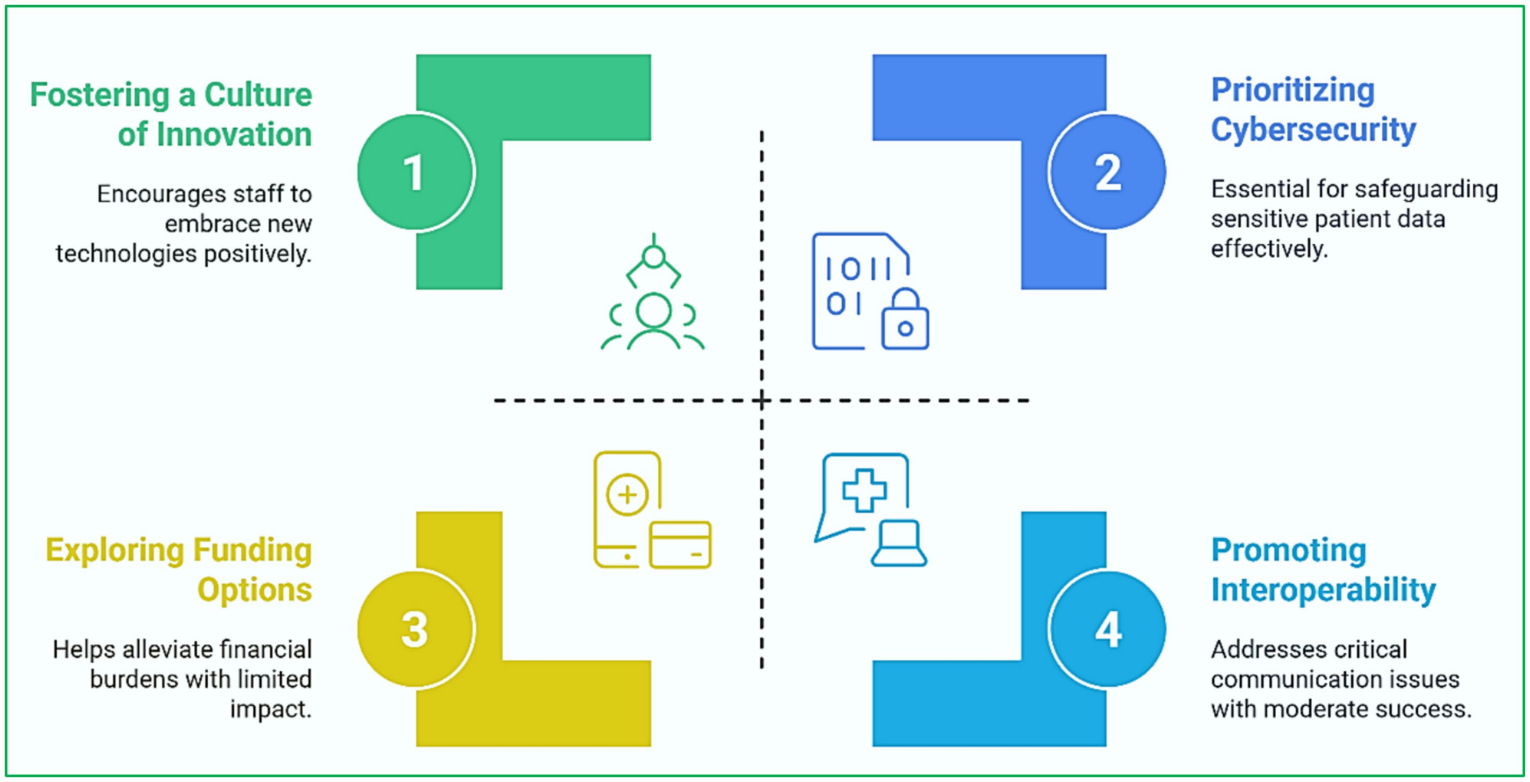
Key challenges in healthcare technology adoption: barriers and strategic solutions.

AI, ML, and emerging technologies hold significant promise to redefine healthcare. However, they must first overcome challenges related to data quality, ethical issues, and clinical integration. These challenges will be prerequisites in determining how best to utilize these technologies for the optimal benefit of both patients and providers. Figure 12 highlights the primary challenges healthcare organizations face in adopting technology and the associated strategic solutions. It identifies key obstacles such as resistance to change, data security challenges, interoperability problems, and significant implementation costs, matched with proactive strategies that encourage an innovative culture, emphasize cybersecurity, support standardized interoperability, and consider alternative funding options. This organized overview underscores that effective integration of digital technologies in healthcare necessitates a comprehensive approach that tackles both technical and human aspects.

## 12 Future directions and emerging trends in ML-based heart disease prediction

### 12.1 Emerging trends in healthcare technology

The healthcare sector is experiencing rapid developments in several areas. It is expected to revolutionize how healthcare is delivered (Junaid et al., 2022). Some of these emerging trends include:

#### 12.1.1 Personalized medicine

Faster healthcare delivery is now achievable due to technological progress in genomics, biomarker discovery, and AI analytics. With the implementation of IoT and ML in healthcare, there is a drastic improvement in diagnosis and care delivery, such as early kidney disease detection and proactive precision care (Loncaric et al., 2020). With the adoption of IoT, subtle risk factors were captured in real-time to assist an ML algorithm that supported personal risk models to provide timely interventions. Therefore, the model ensures continuous patient-provider communication is achieved, thus moving towards personalized healthcare while ensuring strong security measures (Ravi et al., 2025).

#### 12.1.2 AI-driven diagnostics

AI and ML focus on improving the accuracy and timing of diagnosing diseases. From analyzing medical imaging like tumor scanning and cardiovascular monitoring to forecasting patient health through EHR logs, the precision of medicine is improved by automation. In the long term, AI promises to become a ready aid for quick and efficient diagnosis (Sufian et al., 2024; Yenurkar et al., 2024). This research utilizes SVM algorithms to distinguish between types of diabetes, Type 1 and 2, using linear, polynomial, and sigmoid functions. It enhanced the degree of classification incidence of diabetes and enabled the formulation of narrower treatment strategies. It showcased the promise that more developed computational methods can have on medicine with AI-driven healthcare management tasks (Suvartha et al., 2024).

#### 12.1.3 Telemedicine and remote monitoring

Incorporating telemedicine services and remote patient monitoring technologies transforms healthcare delivery, especially in low-resource regions or during public health crises. IoT devices, wearables, and telehealth platforms enable patients to be continuously monitored for their vital signs, medical conditions, and treatment responses (Ardeti et al., 2023; Wang et al., 2024). This development seems poised to continue offering more efficient and inexpensive healthcare services. This work investigated the application of IoT and ML to personalized telemedicine systems with the objective of patient health monitoring and prediction for better care delivery. The effectiveness of the ML model was assessed through recall, sensitivity, error rate, F-score, and other measures, which gave insight into prediction accuracy and complexity. The goal was to shift the paradigm of healthcare to support routine health monitoring and timely proactive interventions (Ramalingam et al., 2024).

#### 12.1.4 Quantum computing (QC)

The emergence of QC promises to revolutionize fields like the modeling of complex diseases and genomics, even improving the standards of drug discovery. Quantum computers could simulate complex biological processes and novel therapeutic targets at speed rivaling thousands of classical computers (Ghazi Enad and Abed Mohammed, 2023). Predicting an enormous acceleration of medical research and precision treatment development is reasonable. In this research, two Quantum ML models, VQC and Pegasos QSVC, were implemented to predict lung cancer in smart healthcare systems. They were benchmarked against baseline models by measuring training accuracy and performing statistical analyses. The Pegasos QSVC model achieved the highest classification accuracy of 85% (Munshi et al., 2024b).

#### 12.1.5 Federated learning (FL)

By not sharing sensitive patient information, FL allows training AI models collaboratively without compromising privacy, which helps gain insights. This innovation facilitates collaboration between healthcare institutions for research and model building while adhering to stringent data privacy regulations. As the fear around the security and privacy of healthcare information further increases, FL is a promising solution for creating AI models that require diverse information while ensuring privacy (Otoum et al., 2024). An ensemble FL approach for developing a DL model to classify data streams from distributed Internet of Medical Things (IoMT) environments was presented. Local federated models were deployed on IoMT devices, with ensemble learning performed on the cloud servers. The comparison results indicated that the ensemble federated model outperformed the primary federated models (Arya and Hanumat Sastry, 2022).

#### 12.1.6 Interdisciplinary approaches for enhanced diagnostics

The merging of ML, AI, and contemporary technologies into healthcare has proven exceedingly useful and requires interdisciplinary teamwork. These technologies’ potential is best capitalized with a multidisciplinary approach toward improving diagnostics. This includes:

- Cooperation Between Health Workers and Data Science Specialists: The adoption of AI in healthcare has proven successful through the collaboration of health workers and data science experts. The data science expert optimizes the ML models, and the healthcare expert adds value with their pathophysiology, clinical diagnosis, and therapeutics knowledge. Their collaboration empowers the development of AI-driven tools that are clinically relevant and accurate, hence ensuring enhanced patient and public healthcare.
- Hybridization of Medicine with Engineering: Biomedical engineers and other engineers are responsible for improving and developing new healthcare technology. Wearable devices, medical imaging systems, or diagnostic aids are some examples of other engineering branches that can be integrated. Engineering and medicine can be united to develop better devices or solutions that match the growing demands of patients and healthcare providers.
- Multidisciplinary Data Integration: Healthcare systems that integrate various data sources like EHRs, Genomics, Imaging, Wearables, and even Environmental Data can enhance diagnosis and treatment direction. Such fusion can be accomplished by interdisciplinary teams using integrated platforms that combine the various datasets to create a more wholesome understanding of the patient’s health and enhance the efficacy of AI models.
- Ethics, Policy, and Technology Integration: To solve AI-related problems in healthcare, collaboration with ethics and policy experts should also take place. Data use’s legal, regulatory, and ethical concerns, such as data privacy, bias, and accountability, must be addressed while technology is developed. Multidisciplinary teams will guarantee the realization of AI tools that are technologically accurate, ethically correct, and legally compliant.
- Patient and Community Involvement: Patients should participate in developing and adopting technology. When patients and communities are brought together through interdisciplinary teams, their issues, preferences, and needs will be addressed so that AI and ML technologies are patient-centric and culturally acceptable. This approach affirms that trust in AI is significantly improved within the frameworks in which diverse populations are formed.

To summarize, the advancement of healthcare technology rests on traditional sectors, which lie in ever-increasing exciting practices and cross-cutting collaborations that will improve diagnostics. New technology like AI, QC, and FL is expected to make healthcare more personalized, accessible, and efficient. Additionally, harnessing multidisciplinary learning will ensure that these results are scientifically valid and ethically responsible, resulting in significant health advantages for patients in all parts of the globe.

## 13 Conclusion

This review encompasses a wide range of ML applications for predicting heart disease, organized into five main themes: detection and diagnostics, ML models and algorithms, feature engineering and optimization, new technologies in healthcare, and cross-disease AI applications. The findings indicate that while DL models, especially hybrid CNN-LSTM architectures, tend to surpass traditional methods, the success of any model heavily relies on high-quality data, effective feature engineering, and clinical interpretability.

A rising trend in FL, monitoring systems based on wearables, and XAI practices that are gradually narrowing the gap between research and practical application. Nevertheless, significant challenges remain, such as ensuring model generalizability across different populations, maintaining data privacy, achieving interpretability, and integrating with EHRs.

Future studies should prioritize the practical implementation of AI systems via clinically validated trials, focusing on ethical and regulatory standards, and investigating interoperable, patient-centered AI platforms. Moreover, creating transparent models that healthcare providers can trust is essential for broad acceptance. By tackling these issues, ML-driven tools can transition from experimental concepts to game-changing solutions in cardiovascular healthcare.

### 13.1 Summary of key insights

■ Diagnostic Accuracy: ML and AI have significantly increased accuracy towards diagnosing heart diseases, cancers, and even neurological disorders. The AI systems utilize EHRs, medical imaging, and large datasets alongside sensors to diagnose and improve patient outcomes.
■ Use of Feature Engineering and Its Optimization: Advanced algorithms, GA, PSO, and GWO, have played a crucial role in improving prediction models, which enhances the accuracy with which disease detection can be made. The AI system was optimized for better interpretability and used to determine feature selection, reduce dimensionality, and use other AI-enabled techniques. Moreover, the AI system can select and interpret relevant features, reduce noise, and improve interoperability through Dimensionality Reduction.
■ Emerging Technologies: Healthcare innovation is changing with QC, FL, and the IoT. QC can speed up complex drug discovery and disease modeling. FL alleviates privacy concerns because sensitive information is not expeditiously sent during collaborative model training. Moreover, the application of IoT devices enables proactive fitness healthcare management by providing real-time monitoring.
■ Issues Relating to Data Quality and Ethical Considerations: The availability, quality, and privacy of healthcare data affect AI implementation in healthcare. The success of AI tools hinges on the availability of accurate and secure healthcare data. Other ethical issues, such as consent, privacy, and the algorithms’ impartiality raisers, must also be addressed for AI to properly deploy in healthcare.
■ Interdisciplinary Collaboration: Collaboration between healthcare personnel, such as doctors and nurses, data analysts and scientists, engineers, and ethics specialists, is essential for creating and implementing AI systems in healthcare. Interdisciplinary approaches ensure that AI technologies are technically sound and respond ethically and socially to the complex conditions surrounding patients and legal affairs.

### 13.2 Implications for research and practice

- *Research:*
  Significant work is currently being done to optimize AI model performance and data assimilation and address ethical concerns. Improving the quality and representativeness of available healthcare datasets will enhance the generalizability of various AI models across different healthcare populations. Further research into other technologies, such as QC and FL, could contribute to advancing AI systems in healthcare. The lack of transparency and interpretability of AI models should also be a focus of future research to foster trust and facilitate smoother integration into clinical decision-making.
- *For Practice:*
  Older healthcare professionals and service providers need to adapt AI and ML-based technologies into their clinical routines to ensure improved diagnostic and treatment results. Sufficient instruction on working with AI systems must be provided alongside the implementation of AI tools to clinicians. Patients should also actively participate in incorporating the technologies so that the solutions work for them. Organizations providing healthcare must also coordinate in fostering interdisciplinary cooperation for the safe, ethical, and beneficial application of AI systems.

AI and ML can transform the health sector, but significant challenges remain in data management, ethics, and deployment. Responsible interdisciplinary collaboration facilitated through innovation has the power to surpass these challenges, which could result in improved health care globally through better utilization of these technologies.

## Glossary

ASVM: Acoustic Support Vector Machine
ADASYN: Adaptive Synthetic Sampling
aTIG-FS: Adaptive Threshold Information Gain Feature Selection
AUC: Area Under the Curve
AI: Artificial Intelligence
ANN: Artificial Neural Network
ABCM: Attention-Based Cross-Modal Transfer Learning
Bi-LSTM: Bidirectional Long Short-Term Memory
CVD: Cardiovascular Disease
CNN: Convolutional Neural Network
DT: Decision Tree
DCGAN: Deep Convolutional Generative Adversarial Network
DL: Deep Learning
ENN: Edited Nearest Neighbor
ECG: Electrocardiogram
EHR: Electronic Health Record
XAI: Explainable Artificial Intelligence
XGB: Extreme Gradient Boosting
FL: Federated Learning
FDA: Food and Drug Administration
GDPR: General Data Protection Regulation
GANs: Generative Adversarial Networks
HIPAA: Health Insurance Portability and Accountability Act
KNN: K-Nearest Neighbor
LSTM: Long Short-Term Memory
ML: Machine Learning
NB: Naive Bayes
PCG: Phonocardiogram
PTB-XL: Physikalisch-Technische Bundesanstalt Extended ECG Dataset
PCA: Principal Component Analysis
QC: Quantum Computing
QSVC: Quantum Support Vector Classifier
RF: Random Forest
ROC: Receiver Operating Characteristic
RNN: Recurrent Neural Network
RFE: Recursive Feature Elimination
RBM: Restricted Boltzmann Machine
SCSO: Sand Cat Swarm Optimization
SHAP: SHapley Additive exPlanations
SVM: Support Vector Machine
SMOTE: Synthetic Minority Over-sampling Technique
VQC: Variational Quantum Classifier
WBAN: Wireless Body Area Network
