# Supplementary figures and images for: A comprehensive review of machine learning for heart disease prediction: challenges, trends, ethical considerations, and future directions
Source: Front Artif Intell. 2025 May 13;8:1583459. doi: 10.3389/frai.2025.1583459 (PMC12106346; doi:10.3389/frai.2025.1583459)

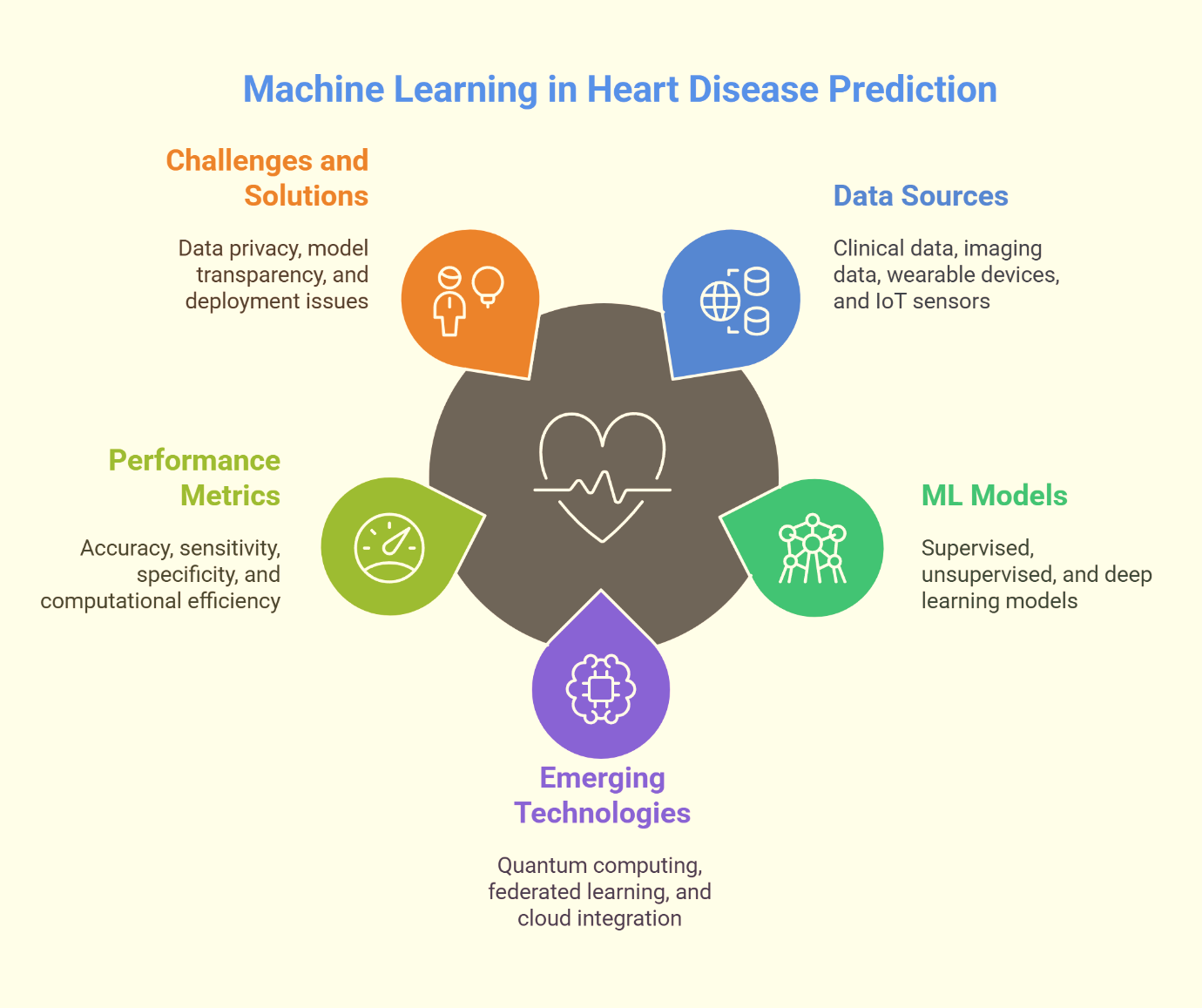

Supplement: Supplementary file 1 [file Data_Sheet_1.docx]
